# Optimal Dielectric Boundary for Binding Free Energy Estimates in the Implicit Solvent

**DOI:** 10.1021/acs.jcim.4c01190

**Published:** 2024-12-10

**Authors:** Negin Forouzesh, Fatemeh Ghafouri, Igor S. Tolokh, Alexey V. Onufriev

**Affiliations:** † Department of Computer Science, California State University, Los Angeles, California 90032, United States; ‡ Genetics, Bioinformatics, and Computational Biology, Virginia Polytechnic Institute & State University, Blacksburg, Virginia 24061, United States; § Department of Computer Science, Virginia Polytechnic Institute & State University, Blacksburg, Virginia 24061, United States; ∥ Department of Physics, Virginia Polytechnic Institute & State University, Blacksburg, Virginia 24061, United States; ⊥ Center for Soft Matter and Biological Physics, Virginia Polytechnic Institute & State University, Blacksburg, Virginia 24061, United States

## Abstract

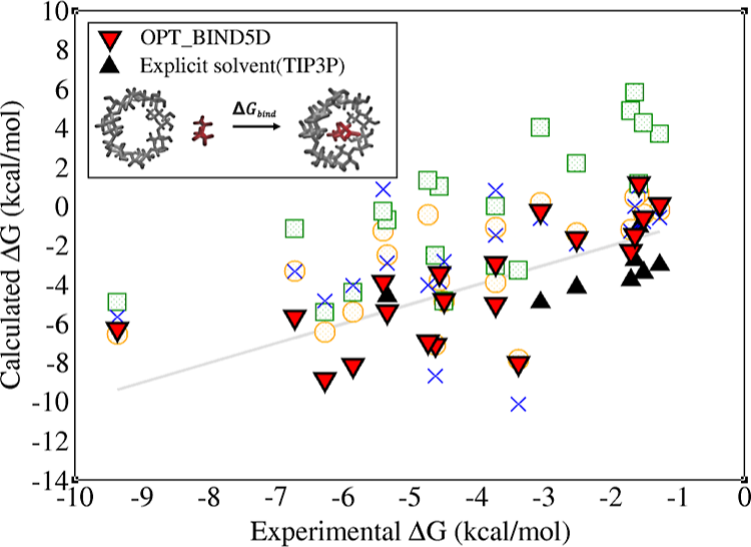

Accuracy of binding free energy calculations utilizing implicit solvent models is critically affected by parameters of the underlying dielectric boundary, specifically, the atomic and water probe radii. Here, a multidimensional optimization pipeline is used to find optimal atomic radii, specifically for binding calculations in the implicit solvent. To reduce overfitting, the optimization target includes separate, weighted contributions from both binding and hydration free energies. The resulting five-parameter radii set, OPT_BIND5D, is evaluated against experiment for binding free energies of 20 host–guest (H–G) systems, unrelated to the types of structures used in the training. The resulting accuracy for this H–G test set (root mean square error of 2.03 kcal/mol, mean signed error of −0.13 kcal/mol, mean absolute error of 1.68 kcal/mol, and Pearson’s correlation of *r* = 0.79 with the experimental values) is on par with what can be expected from the fixed charge explicit solvent models. Best agreement with the experiment is achieved when the implicit salt concentration is set equal or close to the experimental conditions.

## Introduction

Many cellular processes, such as signal transduction, gene expression, and protein synthesis, are governed by the binding interactions of the biomolecules. In structure-based drug discovery, the accuracy and computational efficiency of in silico binding free energy predictions for small molecules to biomolecular targets are crucial for high-throughput screening of potential drug candidates.^1−5^

Achieving fast and accurate computational predictions of binding free energies remains a challenging task,^6−15^ with outcomes heavily dependent on the molecular modeling techniques used, particularly the approximation of the solvent effects.^13,16^ Indeed, conformational transitions involving large changes in the solvent exposed area, such as in protein–ligand binding, are expected to be particularly sensitive to the solvent model used in the computation.^17^

Accuracy limitations are arguably the main concern: it would certainly be desirable for free energy estimates to be within *k*_B_*T* of experiment consistently, but that is not yet the case. Also, to be competitive, the computation should be “fast and accessible” compared to the corresponding experimental effort. Efforts to improve both accuracy and efficiency of various aspects of computational binding prediction are ongoing.^12,15,18−27^ Several major sources of error separate the reality from computation in this area.^28^ Quality of the entire force-field is one of them, of which quality of the solvent model, the main goal of this work, is an integral part.

Two primary categories of solvent models are employed in computational estimates of various aspects of protein–ligand binding: explicit and implicit models. In the explicit solvent framework,^29,30^ each water molecule’s mechanistic details and energetic effects are approximated explicitly, resulting in substantial computational costs, even for arguably the most efficient, fixed-charge, rigid models. Explicit treatment with additional physical effects such as electronic polarizability typically further increases the associated computational costs. The implicit solvent models,^31−35^ which treat the solvent as a continuum dielectric with both polar and nonpolar properties of water, often strike a good balance between accuracy and computational speed. Within this framework, the generalized Born (GB) model^36−45^ is widely used for its simplicity and efficiency.^46,47^

A wide range of methods to estimate free energy of binding exist; see recent reviews.^16,48^ The implicit solvation is particularly well-suited for the generation of conformational ensembles in general and for the so-called “end point” methods: the latter consider only two states, the bound and unbound, and omit the intermediate ones, which makes these methods particularly attractive from the efficiency standpoint. Among end point methods, molecular mechanics generalized Born surface area (MM-GBSA) [or molecular mechanics Poisson–Boltzmann surface area (MM-PBSA)] is a widely used approach,^49−54^ which yields reasonably good correlation of computed Δ*G* with experiment.^48,55^ MM-GBSA (or MM-PBSA) relies on the implicit solvation GB [or Poisson–Boltzmann (PB), respectively] model to estimate the key components of the binding energy.

A key step in the implicit solvent modeling is determining the solute/solvent dielectric boundary (DB)—a region of space over which the dielectric constant ϵ(***r***) changes from the value characterizing the solute interior (e.g., 1) to that of the solvent (e.g., ∼80 for water). The outcomes of implicit solvent calculations are extremely sensitive to the fine details of the geometry and position of the DB,^56,57^ which are determined by the radii of the atoms comprising the solutes (protein and ligand) as well as the water probe radius.^57,58^ Treating the radii as free parameters, optimization of the DB, considering only the four most abundant atom types in proteins (C, H, N, and O) along with the water probe radius, would require minimizing the relevant objective function in a five-dimensional (5D) parameter space. In the past, such optimizations for the solvation free energies of small molecules were performed to minimize the deviation of the computed target from an accepted reference, either experimental or estimated via the explicit solvent.^59−64^

One potential issue with previously derived optimal radii is that finding the truly global minimum of a nonconvex function with many local minima is a very challenging problem,^65,66^ which leaves open the possibility that a significantly better solution may still be found. However, a truly critical limitation of radii sets optimized against small molecule hydration free energies (HFE) is that these parameters deliver suboptimal accuracy in estimates of binding free energies.^7,67,68^ While more than one reason could be behind the observed deterioration of accuracy, overfitting of the necessarily imperfect practical solvent models to small molecule hydration data is the likely suspect. Indeed, recent successful efforts^12,27^ to refine parameters of a solvent model directly against binding free energies suggest that using the latter as the target of the optimization holds considerable promise. However, using binding energies as the only optimization target holds the risk of overfitting to the necessarily very limited set of targets used in such an effort; the limitations on the number of structures in the training set and their size are expected to be particularly severe as the number of parameters to be optimized grows.

Recently, we have developed a computational protocol^69^ that is capable of optimizing the DB parameters—the atomic radii and water probe radius—directly against the electrostatic component of the binding free energy precomputed in the explicit solvent. For a necessarily relatively small training set of neutral protein–ligand complexes, for which such a computation was feasible, an optimum solution was found for which the resulting agreement with the explicit solvent reference was notably better than for any other existing radii set tested. However, before the approach can be used for practical binding free energy estimates in the implicit solvent, two critical issues must be addressed. First, the parameters of the DB optimal for a limited set of neutral protein–ligand complexes are very likely overfitted for this specific training set. Using a much larger training set of diverse protein–ligand complexes might seem like an obvious solution, but it would be completely out of reach due to the highly demanding nature of the multidimensional optimization in the context of protein–ligand binding. Thus, a different procedure leading to a more generalizable set of radii must be developed. In addition, critically, its outcomes will have to be tested directly against experiment, not just for the electrostatic component but for the entire experimental binding free energy. The goal of this work is to identify and thoroughly test a computationally feasible approach that preserves the advantages of optimizing atomic radii directly within the context of MM-GBSA binding free energy calculations while ensuring that the resulting parameters are not overfitted for the necessarily small training set. We stress that the focus of the work is improving the accuracy of the solvent model used in these kinds of estimates, which determines some of the methodological choices made.

The rest of this paper is organized as follows. We begin in the Materials and Methods section, where we detail the computational techniques and protocols employed in our study. This is followed by the Results and Discussion section, which is divided into two main parts. The first part presents our new strategy for determining optimal radii sets for protein–ligand binding. The second part focuses on evaluating these optimal radii sets. We test their accuracy using host–guest (H–G) systems and explore the effects of salt concentration on the MM-GBSA calculation results. Finally, we conclude with a summary of our findings, discussing the implications of our results and suggesting directions for future research.

## Materials and Methods

### Overview of the Radii Optimization Pipeline

We employ a multidimensional optimization pipeline, previously developed^69^ for finding the optimal DB in protein–ligand binding free energy calculations. Here, we explore the optimization of atomic radii, which form the DB, using a multiterm objective function made of binding free energy and solvation free energy terms. The new optimal radii are tested against experiment in the context of H–G systems using MM-GBSA, which provides an end-state free energy method.

### Optimization in the Space of Atomic and Water Radii

**Figure 1:**
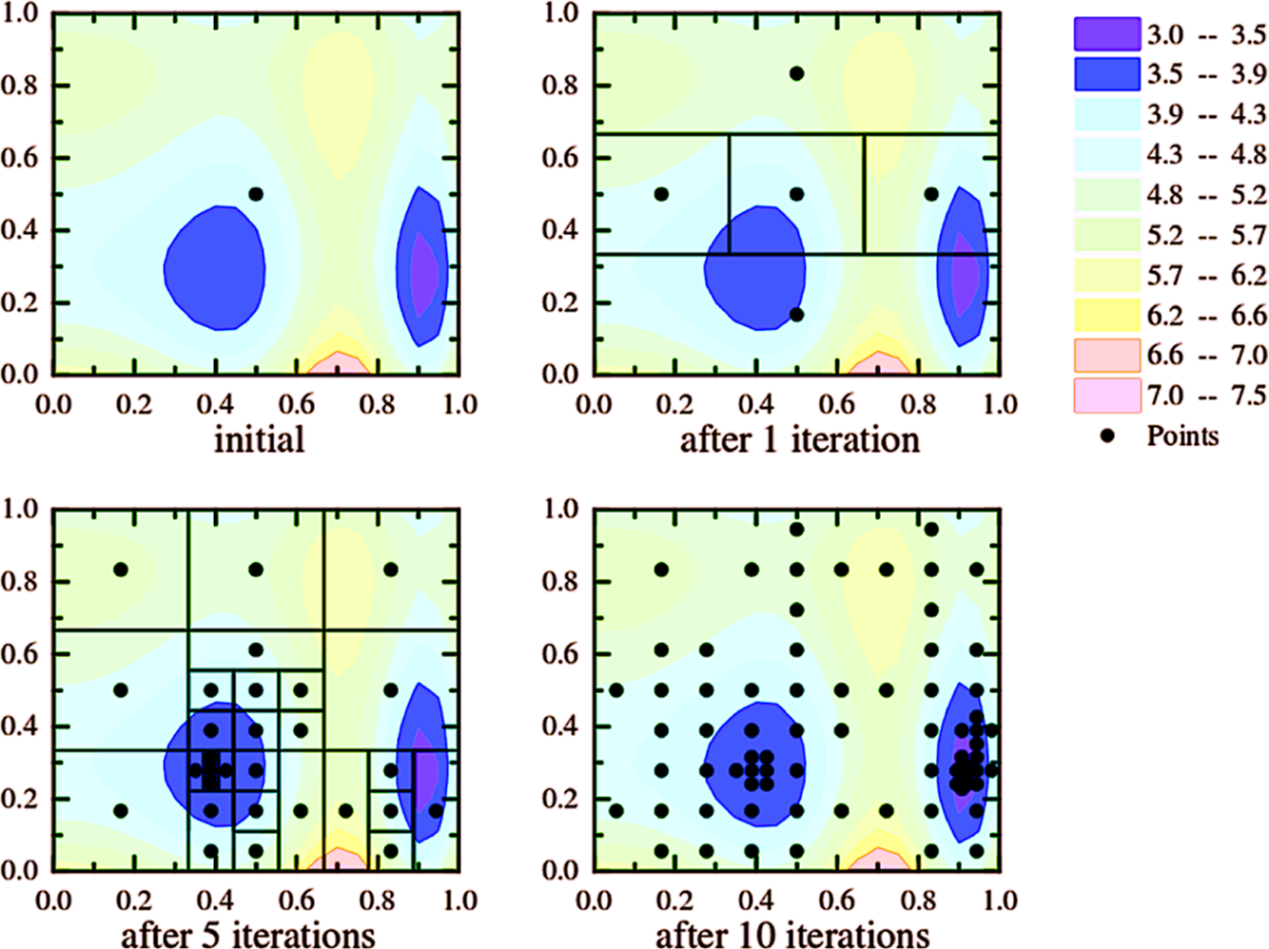
Example of DIRECT algorithm steps. Shown are objective function evaluations performed by the DIRECT search algorithm after 0, 1, 5, and 10 iterations. In this example, the algorithm converges to the global minimum solution rapidly due to the efficient selection method, which only chooses potentially optimal boxes for sampling in the next steps. The values of the objective function (in arbitrary units) are color coded as shown in the inset in the top right corner.

A deterministic search algorithm called DIRECT^70^ is employed in this study, which is remarkably frugal in terms of the number of function evaluations, recommended for relatively low-dimensional problems, i.e., *D* < 100 (*D* is the number of parameters to be optimized). This algorithm does not require knowledge of the Lipschitz constant of the problem. The overall idea of DIRECT is shown in Figure 1.

### Implicit Solvent Model Used

In this work, the polar component, Δ*G*_pol_, of the solvation free energy of a solute, Δ*G*_solv_, is calculated via the GBNSR6 flavor^71−73^ of the GB model,^47^ using the analytical linearized Poisson–Boltzmann (ALPB) approach.^74,75^ The ALPB introduces into the canonical GB model of Still et al.^38^ physically correct dependence on the dielectric constants of solute, ϵ_in_, and solvent, ϵ_out_, respectively.

GBNSR6 has recently been shown to be the most accurate among several other GB flavors in predicting the electrostatic (polar) components of binding free energies, Δ*G*_bind_^pol^, where the results from the PB model were chosen as the reference.^76^ Notice that, while the PB^63,77−84^ is generally more accurate than the GB, using a high-accuracy PB model directly in a powerful multidimensional optimization pipeline for calculating Δ*G*_bind_^pol^ is extremely computationally demanding; in this respect, GBNSR6 provides an acceptable compromise, approximating the PB solvation free energies reasonably well, at a small fraction of the cost.

Within GBNSR6, the solute/solvent DB is represented by a solvent-excluded surface (SES), which enters into the model via the effective Born radii.^47^ To make the calculations even more efficient, we utilize the grid-based implementation of the GBNSR6 model,^73^ which approximates the ideal SES with orthogonal grid patches.^85^ A detailed analysis of this GBNSR6 implementation and its input parameters is provided in ref (73). Here, we use it with the grid resolution set to 0.5 Å (default value). Other details of the optimization protocol and specific parameter settings can be found in ref (54).

### Data Sets for Training, Validation, and Testing

#### Protein–Ligand Complexes: Training and Validation

The entire data set consists of 15 protein–ligand complexes, for which the electrostatic (polar) components, Δ*G*_bind_^pol^, of binding free energies in the TIP3P explicit solvent were previously estimated as described in ref (86). The standard and well-justified^87^ method of free-energy decomposition was used there, where the polar components of binding free energies were estimated using thermodynamic integration by gradually turning on the charges on the solutes after the nonpolar (LJ) solute–water interactions have been equilibrated. The Δ*G*_bind_^pol^ defined this way includes the changes of the gas-phase electrostatic interactions, Δ*E*_el_, in the protein–ligand complexes upon binding: Δ*G*_bind_^pol^ = ΔΔ*E*_el_ + ΔΔ*G*_pol_. This data set was also used in similar contexts in previous works.^76,88^ The complexes are highly diverse with respect to the absolute values of Δ*G*_bind_^pol^ (0.7–25 kcal/mol), making them good candidates for studies aimed at improving computational drug discovery. At the same time, the complexes are relatively small in size (1635–1995 atoms), which proves critical for success of the computationally intense optimization procedure utilized in this work. All proteins and ligands components in the complexes are neutral, or were forced to be neutral; see details in ref (86). The neutrality allows one to avoid various uncertainties and complications due to the use of Ewald summation and periodic boundary conditions in the explicit solvent simulations used in previous study^86^ to estimate Δ*G*_bind_^pol^ employed here as the reference. To mitigate uncertainties due to conformational variability of the proteins, the structures of the complexes were restrained after the relaxation in vacuum.^86^ In order to train and validate the proposed computational protocol, the entire data set is partitioned into Train and Validation subsets: 8 complexes in the Train set [1pbk, 1fkf, 1bkf, 1fkh, 2hah, 2fke, 1zp8, 1f40 (protein PDB IDs^86^)] and 7 complexes in the Validation set (1b11, 1fb7, 1fkb, 1fkg, 1fkj, 1fkl, and 3kfp). This partitioning provides similar distribution of Δ*G*_bind_^pol^ energies between the two subsets.

#### Small Molecules Hydration: Training and Validation

We use a set of nearly rigid small neutral molecules^89^ from the FreeSolv database^90^ initially selected by Mukhopadhyay et al.,^89^ from which we further selected a subset of 173 molecules with only C, H, N, and O atom types. The experimental and the TIP3P explicit solvent HFEs for this subset are available in the FreeSolv database,^90^ with the corrections provided in ref (91). We split the selected rigid molecules subset into two fractions (88 and 85 molecules) to use as our Train and Validation sets of small molecules, respectively. For each of these sets, the polar components, Δ*G*_pol_, of the HFEs are estimated using the GBNSR6^71−73^ flavor of the GB model, and the corresponding root mean square errors, RMSE_solv values (Train/Validation) against the polar components of TIP3P HFEs, are calculated. See details in the Implicit Solvent Model Used subsection above.

#### Combined Training and Validation Sets

We combine 8 protein–ligand complexes and 88 small molecules from the corresponding Train sets, described above, and use their binding and hydration energy data to further optimize (refine, make more robust) the binding model. A complementary combined Validation set consists of 7 protein–ligand complexes and 85 small molecules from the corresponding separate Validation sets.

### Testing against Experiment: Host–Guest Complexes

The H–G systems can now be considered as standard for evaluating and improving computational methods used to predict binding free energies.^12,27,92^ Below is a brief description of the selected H–G systems used in our work, which come from recent benchmark studies.^16,93^ Here, we use these structures *as is*, without any further modifications from the above references. The hosts are relatively small molecules (about 140–220 atoms for the H–G complexes used here) with a binding cavity, which can be used in computational models of noncovalent binding due to their ability to bind guests via forces similar to protein–ligand interactions, while their rigidity facilitates comprehensive conformational sampling, thus mitigating potential errors in the conformational entropy estimates. The latter is particularly important for us here as our goal is to evaluate the accuracy of a solvent model. The utilization of H–G systems is also useful to demonstrate transferability of the newly developed radii sets for binding, since the set is optimized for small protein–ligand complexes. The following set of 20 H–G complexes (Table 1) was selected from the two benchmark data sets:^16,93^ 10 complexes from the octa acids (OA) Gibb deep cavity cavitands (GDCC) and 10 complexes from the cyclodextrins (CD). The selected OA-GDCC systems consist of highly charged hosts, while the selected CD hosts are in low charge or neutral states. The selection process aimed to encompass a broad range of experimental Δ*G*_bind_^0^ values, from nearly −10 to −1 kcal/mol, and to focus on “clean” complexes with high-quality experimental Δ*G*_bind_^0^ available.

**Table 1:** Test Set of Host–Guest Complexes

complex	guest molecule
OA-6(G4)	4-bromoadamantane-1-carboxylic acid
OA-4(G6)	3-nitrobenzoic acid
OA-8(G5)	trimethylphenethylaminium
OA-3(G1)	5-hexynoic acid
OA-5(G2)	4-cyanobenzoic acid
OA-7(G3)	*N*,*N*,*N*-trimethylhexan-1-aminium
OA-4	4-chlorobenzoic acid
OA-1	benzoic acid
OA-2	4-methylbenzoic acid
OA-3	4-ethylbenzoic acid
α-CD-8-p	octanoic acid
α-CD-6′-p	butanoic acid
α-CD-1-p	1-butylamine
α-CD-7-p	hexanoic acid
α-CD-5-p	cycloheptanol
β-CD-9-p	phenylacetic acid
β-CD-6-p	pentanoic acid
β-CD-8-p	benzoic acid
β-CD-5-p	cycloheptanol
β-CD-3-p	cyclopentanol

The input structures of the H–G complexes, including force-field parameters and partial charges, were originally prepared as detailed in refs (16 and 93). For readers’ convenience, we summarize below the key steps of the corresponding preparation protocols from the above references:

(1) First set of 10 H–G systems: OA-GDCC hosts

The molecular structures of free host OA, along with unbound guests, were manually constructed, and quantum mechanics (QM) energy was minimized using the HF/6-31G(d) method in Gaussian 09. Net charges of −8*e* were assigned to OA hosts to reflect their protonation states in the experimental basic pH conditions. Simulations utilized GAFF^94^ v1.7 parameters for bonded and LJ interactions, while partial charges were assigned via the RESP approach. Starting bound configurations were generated through docking, followed by solvation in a cubic box with 2100 TIP3P water molecules. Counterions (Na^+^ or Cl^–^ ions) were added only for neutralization and were modeled using TIP3P-specific sodium parameters. Following equilibration, a 2 ns *NVT* simulation (simulation input) yielded a frame with the most populated configuration via clustering.

(2) Second set of 10 H–G systems: CD hosts

The structures for unbound α- and β-CD, alongside guest molecules, were manually constructed. Guest molecules underwent vacuum-based QM energy minimization using the HF/6-31G(d) method within Gaussian 09. Partial charges, LJ parameters, and bonded parameters were derived from Q4MD-CD force fields for CD molecules.^95^ Guest partial charges were derived using the RESP method implemented in the R.E.D. server tool,^96^ while LJ and bonded parameters were sourced from GAFF^94^ v1.7. Molecular dynamics (MD) simulations of these systems included the H–G complexes, TIP3P waters, and additional Na^+^ and Cl^–^ ions for neutralization, approximating the ionic strength of a 50 mM phosphate buffer used in experiments. Equilibration with positional restraints was conducted. The approach accommodated guest orientations within CD cavities using designated simulation files. Equilibration in the *NPT* ensemble with light positional restraints (0.1 kcal/mol/Å^2^) on host and guest atoms was performed, and the equilibration’s final conformation was retained. Unrestrained equilibration and clustering were omitted for CD sets, considering that certain guest bindings might be weak enough to permit extended departures from the binding cavity. For accommodating guest’s potential orientations within the CD cavity, simulation files marked with the “-p” suffix signify that the guest molecule is linked with its polar functional group positioned outward from the primary (narrow) side of the CD structure.

#### MD Simulations of Host–Guest Systems

All of the MM-GBSA estimates for H–G systems are based on the snapshots extracted from the MD trajectories of these systems generated as described below. The structures were prepared and equilibrated as follows. The solvated complexes, using the TIP3P water model, were first energy minimized (max. minimization cycle of 1000), followed by 50 ps of heating (from 0 to 300 K) at constant volume, followed by 50 ps of density equilibration at 300 K at constant 1 bar pressure. In these stages, residues of both the host and guest molecules were restrained to their initial positions with a force constant of 2 kcal/mol/Å^2^. These stages are followed by another 100 ns of constant pressure (1 bar) equilibration at 300 K with no restraints. All simulations, including the production runs described below, were executed with the GPU-enabled *pmemd.cuda* engine in AMBER20 and AMBER22, using Langevin dynamics with a collision frequency of 1 ps^–1^ and an integration time step of 2 fs. The bonds involving hydrogen atoms were constrained by the SHAKE algorithm. Electrostatic interactions were approximated via the particle mesh Ewald method, with a nonbonded cutoff set to 9 Å. Unless otherwise stated, the length of the production phase in each MD simulation was 1 μs. The snapshots with coordinates of the system were recorded at 1 ns intervals (each 5,00,000 time steps).

#### Estimating Δ*G*_bind_^0^ Directly from Binding–Unbinding Events

There is a subset of our H–G systems with relatively low binding affinities, such that during our 1 μs MD simulations in the explicit solvent, the guest molecule enters and exits the host pocket many times, forming unbound states observed in multiple frames. We call this subset of the complexes the “low affinity” subgroup; see Table S4. To eliminate any potential image artifacts, a careful visualization of the MD trajectories is conducted following autoimaging procedures.

For this (and only this) subgroup of H–G systems, the standard free energy of binding, Δ*G*_bind_^0^, is calculated employing the following equation:^97,98^
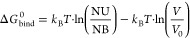
1Here, NU is the number of frames/snapshots in which the guest and host are unbound, while NB corresponds to the count of frames/snapshots in which they are bound. To account for the simulated system’s volume, *V*, being different from the standard state volume, *V*_0_ = 1, 660 Å^3^ [at *T* = 298 K and 1 M (1 mol/L) H–G system concentration],^97^ an entropy correction to the free energy of binding, the second term in the above equation, is applied. Regarding the correction values: for the OA systems simulated within a 41 × 41 × 41 Å^3^ cubic box and the CD systems—within a 36 × 36 × 36 Å^3^ box, the corrections are −2.21 and −1.98 kcal/mol, respectively.

**Figure 2:**
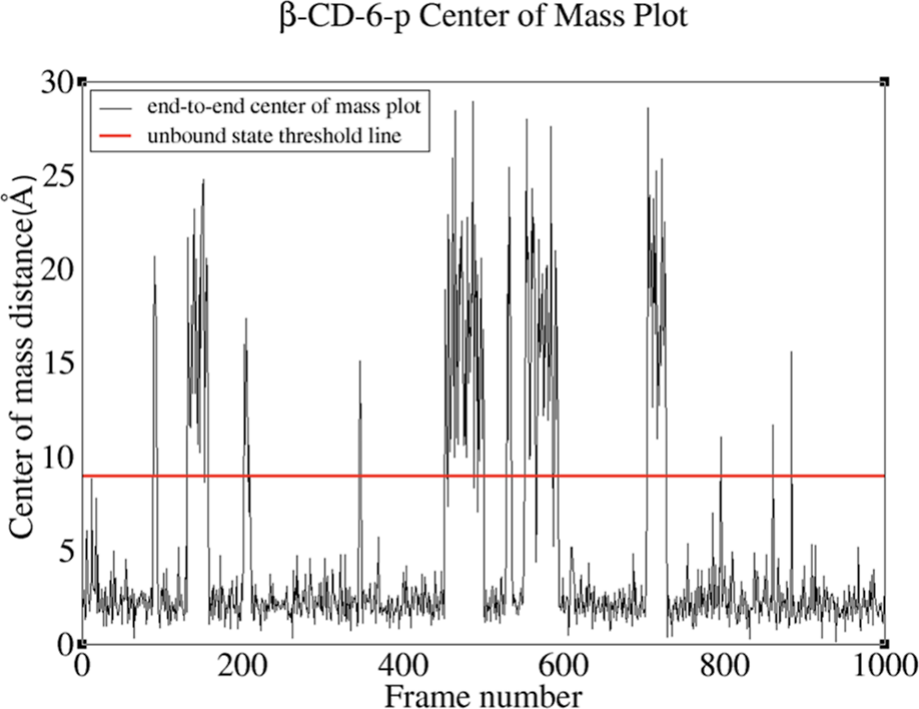
Variation of the distance between the H and G centers of mass in the β-CD-6-p H–G system along the system trajectory. The β-CD-6-p system is used as a representative of the low-affinity complexes. The *Y*-axis represents the distance from the center of mass of the guest molecule to the host molecule center of mass. The *X*-axis represents the frame index along the system trajectory. The distances are obtained using the *cpptraj* tool in the AMBER package. The red line indicates the calculated threshold value of 9 Å for this system to distinguish bound and unbound states.

To estimate the number of NU and NB frames, we first used the *cpptraj* tool in the AMBER package to determine the distance between the host and guest centers of mass in each frame of the H–G trajectory (Figure 2). Then, to separate bound and unbound states in different H–G systems, a threshold distance between these centers of mass is defined dynamically as the sum of the host and guest radii of gyration (red line in Figure 2), calculated using the AMBER *cpptraj* tool. Significantly, this classification method aligns with observations made using Visual Molecular Dynamics,^99^ a widely used molecular visualization tool, achieving a consistency rate of approximately 95%. This high rate of agreement shows the reliability of the methodology in distinguishing bound from unbound states of H–G systems. We have also tested the robustness of the explicit solvent estimates of Δ*G*_bind_^0^, eq 1, with respect to the threshold value that separates the bound and unbound states (Figure 2). The analysis shows that within a 2 Å range above or below the current threshold value, the free energy of binding estimates varies by less than 7%.

#### Robustness of Δ*G*_bind_^0^ to Trajectory Length and Number of Snapshots Used

To verify whether a 1 μs duration of MD simulations with the explicit solvent suffices for our objectives in estimating Δ*G*_bind_^0^ directly from binding–unbinding events using eq 1, we selected the β-CD-6-p H-G system, which has the weakest experimental binding affinity (−1.27 kcal/mol) among all the entries in our data set. This choice makes it particularly susceptible to inevitable methodological inaccuracies. Consequently, we conducted a 3 μs MD simulation of this H–G system and subsequently recalculated the binding energy using eq 1. We also assessed the effect of the snapshot sampling frequency on the resulting Δ*G*_bind_^0^ estimates using original 1 μs long MD simulation of the β-CD-6-p system. To do this, we decreased the snapshot sampling from every 1 ns to every 2 ns. The results (calculated Δ*G*_bind_^0^ values) are presented and discussed in Table 5 in the Results and Discussion section.

**Table 2:** Optimized Sets of Water Probe and Atomic Radii (Å) and Related RMSE Values (kcal/mol) Corresponding to Different Weights (w1:w2) of the RMSEs for the Polar Components of the Solvation Free Energies of Small Molecules (RMSE_solv) and Binding Free Energies of Protein–Ligand Complexes (RMSE_bind) in the Objective Function: RMSE_total = w1 × RMSE_solv + w2 × RMSE_bind^a^

radii set (w1:w2)	OPT_BIND (0:1)	OPT_BIND (0.1:1)	OPT_BIND (0.3:1)	OPT_BIND (1:1)	OPT_SOLV (1:0)
ρ_W_	1.37	1.35	1.35	1.35	0.53
ρ_C_	1.40	2.23	2.23	2.25	1.92
ρ_H_	1.55	1.47	1.47	1.48	1.29
ρ_N_	2.35	2.37	2.37	1.75	1.72
ρ_O_	1.28	1.09	1.09	1.11	1.74
Train set					
RMSE_solv	[2.30]	2.14	2.14	1.80	0.83
RMSE_bind	3.94	4.60	4.60	4.74	[20.61]
RMSE_total	3.94	4.81	5.24	6.54	0.83
Validation set					
RMSE_solv	2.65	2.48	2.48	1.98	0.97
RMSE_bind	6.62	4.99	4.99	5.59	22.01
RMSE_total	6.62	5.24	5.73	7.57	0.97

a The objective function is minimized using combined Train set of molecules. RMSE values over Validation set of molecules are presented as well. RMSE values shown in the square brackets were not used in the optimization (due to zero weights in the objective functions) and are estimated for comparison purpose only. Note that OPT_SOLV (1:0) is equivalent to OPT_BIND (1:0)—the radii set optimized exclusively against polar HFEs of small molecules.

**Table 3:** Comparison of the New Radii Sets [OPT_BIND (0:1) and OPT_BIND5D] and Predicted RMSE Values (kcal/mol) with Other Common Radii Sets (BONDI, PARSE, and ZAP-9) and the Corresponding RMSE Values, over the Train and Validation Sets of Molecules^a^

	BONDI	PARSE	ZAP-9	OPT_BIND (0:1)	**OPT_BIND5D**
ρ_W_	1.40	1.40	1.40	1.37	**1.35**
ρ_C_	1.70	1.70	1.87	1.40	**2.23**
ρ_H_	1.20	1.00	1.10	1.55	**1.47**
ρ_N_	1.55	1.50	1.55	2.35	**2.37**
ρ_O_	1.50	1.40	1.52	1.28	**1.09**
ρ_S_	1.80	1.85	2.15	1.80	**1.80**
Train set					
RMSE_solv	2.11	3.55	1.86	2.30	**2.14**
RMSE_bind	7.62	10.19	7.31	3.94	**4.60**
RMSE_total	7.83	10.54	7.49	4.17	**4.81**
Validation set					
RMSE_solv	2.04	3.38	1.72	2.65	**2.48**
RMSE_bind	8.72	7.93	8.30	6.62	**4.99**
RMSE_total	8.92	8.27	8.47	6.89	**5.24**

a The radii are in Å. All RMSE_total values are calculated for (0.1:1) ratio of RMSE_solv and RMSE_bind contributions as were the weights in the objective function for the OPT_BIND5D radii set.

**Table 4:** Accuracy of the Δ*G*_bind_^0^ Estimates across the Entire Data Set of 20 H–G Systems Relative to Experiment^a^

radii set	RMSE	*r* correlation	MSE	MAE	SRC
mbondi	2.37	0.70	1.16	1.92	0.68
PARSE	4.46	0.82	3.79	3.82	0.78
OPT_BIND (0:1)	2.96	0.51	1.28	2.36	0.61
**OPT_BIND5D**	**2.03**	**0.79**	**–0.13**	**1.68**	**0.83**

a The estimates are calculated for the 0.154 M salt concentration. The errors (RMSE, MSE, and MAE) are in kcal/mol. Spearman’s Rank Correlation Coefficient (SRC) is the r correlation coefficient calculated using the ranks of the data instead of their actual values.

**Table 5:** Analyzing the Consistency of Δ*G*_bind_^0^ Values (in kcal/mol) for the β-CD-6-p H–G System Calculated Directly from Eq 1, Using the Original (1 μs) and Longer (3 μs) Simulations in TIP3P Water^a^

simulation/frames	simulation time	NU	NB	binding/unbinding events	Δ*G*_bind_^0^in TIP3P
original/1000 frames	1 μs	162	838	25	–2.97
original/500 frames	1 μs	79	421	25	–3.04
long/3000 frames	3 μs	499	2501	102	–2.83

a The total number of frames (NU + NB) used to estimate Δ*G*_bind_^0^ are shown in the left column. The trajectory of the original simulation (1000 frames, 1 μs) is shown in Figure 2.

#### MM-GBSA Calculations

Single trajectory MM-GBSA calculations were performed to estimate the binding free energies in H–G complexes
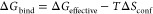
2where the effective binding energy, Δ*G*_effective_,^100,101^ is calculated by accounting for the changes in the gas-phase energies [Lennard-Jones (Δ*E*_LJ_) and electrostatic (Δ*E*_el_) interactions] and in the polar, Δ*G*_pol_, and nonpolar, Δ*G*_np_, contributions to the solvation free energies, Δ*G*_solv_, resulting in
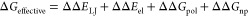
3where ΔΔ indicates a change of the corresponding contribution upon binding.^69^ The entropy term (−*T*ΔS_conf_) in eq 2 is the contribution to Δ*G*_bind_ due to the changes in the configurational entropy, *S*_conf_, of solutes (both of the receptor and the ligand) upon binding. Note that this term does not exactly correspond to the experimental entropy contribution (−*T*Δ*S*) because within the implicit solvation approach, the entropy changes due to solvent rearrangement are already incorporated into ΔΔ*G*_pol_, which is a part of Δ*G*_effective_. Likewise, Δ*G*_effective_ does not correspond directly to experimental Δ*H*_bind_. The computations of the gas-phase energies, Δ*E*_LJ_ and Δ*E*_el_, and the nonpolar components (Δ*G*_np_) of solvation free energies are executed utilizing the mm_pbsa.pl module within the AMBER18 software suite (AmberTools18). A Python implementation of this module, MMPBSA.py, is also available in AmberTools18 and later versions.^102^ The nonpolar contributions to the solvation free energies are calculated as
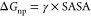
4and are proportional to the solvent-accessible surface areas (SASA) of molecules and to the surface tension constant, γ. Atomic radii that, together with a water probe radius, determine that SASA are important for both nonpolar and polar components (through the geometry of dielectric boundary, DB/SES), which highlights the importance of the right choice of atomic radii for the accuracy of binding free energy calculations.^103,104^ The polar components (Δ*G*_pol_) of the solvation free energy are computed using a grid-based surface GB model known as GBNSR6.^71−73^ Starting with AmberTools24, the default GB model used in the MMPBSA.py module is GBNSR6 (igb = 66). It is widely known that GB models are typically less computationally demanding than PB models. However, there remains a consistent concern regarding the potential decline in predictive accuracy associated with GB models.

Through the MM-GBSA approach, the averages of Δ*G*_pol_ and Δ*G*_np_ are calculated on a collection of structural ensembles (snapshots) extracted from the MD simulation. In this context, a total of 1000 snapshots were systematically sampled every 1 ns over the course of a 1 μs (unless otherwise specified) MD trajectory for each H–G system. IGB and GBSA parameters were set to 66 and 6, respectively, to implement GBNSR6.^71^ The dielectric constants for the solvent and the solute were set to 80 and 1, respectively. Following previous studies,^19^ we calculate Δ*G*_bind_ at physiological salt conditions, assuming 0.154 M salt concentration.^105^ To calculate Δ*G*_np_ with no additional offset, γ was set to the default value,^64^ 0.005 kcal/mol/Å^2^.

Entropy (Δ*S*_conf_) calculations were also performed using the mm_pbsa.pl module in the AMBER18 software package. Normal-mode analysis was chosen over quasi-harmonic analysis for calculating configurational entropy due to its better convergence properties.^100^ To enable entropy decomposition using the “original” nmode implementation, the PROC parameter was set to 2. The MAXCYC parameter was set to 1000 minimization cycles to ensure convergence. To ensure that the energy optimization reaches a stable state, the convergence threshold, the DRMS parameter, was set to 0.5. By setting IGB to 0, vacuum electrostatics was used instead of the GB model. The dielectric constant for the solvent was set to 80, and the effect of 0.154 M monovalent salt was treated implicitly,^47^ at the Debye–Hükel level. The surface tension constant was set to the value^38^ of 0.0072 kcal/mol/Å^2^ and, finally, the DIELC parameter was set to 4 to specify a distance-dependent dielectric constant as the IGB was already set to 0. An offset of 1.92 kcal/mol has been subtracted from the—*T*Δ*S*_conf_ component of GBNSR6 calculations to adjust the translation entropy estimate between the standard conditions in the gas phase vs in the solution; see refs (98 and 100) for details. The resulting Δ*G*_bind_ becomes Δ*G*_bind_^0^, which facilitates direct comparison with typical experimental data. Incorporating the entropy offset contributes to the enhanced accuracy of the calculations. Numerical values of the components of Δ*G*_bind_, eqs 2–4, are available in the Supporting Information.

#### Bootstrap Estimation of Standard Errors and Confidence Intervals

For all the performance metrics reported in Table 6, we employed bootstrap resampling techniques to estimate the standard errors.^106^ Specifically, we developed a Python script to perform 1000 bootstrap iterations with replacement. The 95% confidence intervals were calculated based on these bootstraps using the following equation for the confidence interval^107^
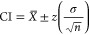
5Here, *X̅* represents the sample mean, σ is the standard deviation of the bootstrap samples, *z* is the critical value from the standard normal distribution corresponding to the 95% confidence level (approximately 1.96), and *n* is the sample size.

**Table 6:** Accuracy of the Δ*G*_bind_^0^ Estimates against Experiment, Computed for Different Subgroups in the H–G Test Set and for Different Radii Sets^a^

	all	highly charged	near neutral	low affinity
	20 complexes	10 complexes	10 complexes	8 complexes
mbondi				
RMSE	2.37 ± 0.13	2.45 ± 0.19	2.27 ± 0.17	2.01 ± 0.14
*r* correlation	0.70 ± 0.06	0.70 ± 0.08	0.86 ± 0.06	0.80 ± 0.15
MSE	1.16 ± 0.20	1.76 ± 0.24	0.57 ± 0.29	1.80 ± 0.14
MAE	1.92 ± 0.13	1.90 ± 0.21	1.95 ± 0.15	1.79 ± 0.13
SRC	0.68 ± 0.06	0.74 ± 0.09	0.73 ± 0.08	0.47 ± 0.15
PARSE				
RMSE	4.46 ± 0.19	3.52 ± 0.24	5.22 ± 0.25	5.69 ± 0.20
*r* correlation	0.82 ± 0.03	0.57 ± 0.07	0.77 ± 0.07	0.78 ± 0.15
MSE	3.79 ± 0.22	2.84 ± 0.28	4.72 ± 0.31	5.50 ± 0.22
MAE	3.82 ± 0.22	2.91 ± 0.27	4.71 ± 0.31	5.50 ± 0.22
SRC	0.78 ± 0.05	0.59 ± 0.11	0.60 ± 0.10	0.29 ± 0.17
OPT_BIND (0:1)				
RMSE	2.96 ± 0.25	3.09 ± 0.26	2.82 ± 0.33	1.70 ± 0.13
*r* correlation	0.51 ± 0.07	0.75 ± 0.07	0.82 ± 0.06	0.83 ± 0.12
MSE	1.28 ± 0.29	2.64 ± 0.21	–0.09 ± 0.40	1.45 ± 0.14
MAE	2.36 ± 0.21	2.63 ± 0.21	2.06 ± 0.26	1.45 ± 0.14
SRC	0.61 ± 0.09	0.80 ± 0.10	0.77 ± 0.08	0.62 ± 0.12
**OPT_BIND5D**				
RMSE	**2.03** ± 0.12	**1.85** ± 0.11	**2.19** ± 0.20	**1.55** ± 0.16
*r* correlation	**0.79** ± 0.04	**0.55** ± 0.10	**0.79** ± 0.06	**0.89** ± 0.16
MSE	**–0.13** ± 0.20	**–0.52** ± 0.24	**0.23** ± 0.31	**1.03** ± 0.17
MAE	**1.68** ± 0.11	**1.64** ± 0.12	**1.77** ± 0.17	**1.19** ± 0.15
SRC	**0.83** ± 0.04	**0.68** ± 0.09	**0.78** ± 0.07	**0.64** ± 0.13
Explicit solvent (TIP3P)				
RMSE	N/A	N/A	N/A	1.67 ± 0.07
*r* correlation	N/A	N/A	N/A	0.66 ± 0.07
MSE	N/A	N/A	N/A	–1.25 ± 0.17
MAE	N/A	N/A	N/A	1.57 ± 0.08
SRC	N/A	N/A	N/A	0.90 ± 0.06

a The estimates are calculated for 0.154 M monovalent salt concentration. RMSE, mean signed error (MSE), and mean absolute error (MAE) values are in kcal/mol. The root-mean-square difference (RMSD) between the MM-GBSA results for Δ*G*_bind_^0^ and explicit solvent values (calculated directly from binding/unbinding events) for the low-affinity group is approximately 2.70 kcal/mol. The error bar represents 95% bootstrap confidence intervals; see Materials and Methods.

## Results and Discussion

### New Strategy for Finding the Optimal Radii Set for Protein–Ligand Binding

Here, we explore the following strategy for finding a set of intrinsic atomic radii that, by determining a geometry of the solute/solvent DB, yields the most accurate binding free energies and can be used with the MM-GBSA (implicit solvent) model. The objective function we minimize over a set of atomic radii is a weighted mix of the RMSEs relative to the reference binding energies, RMSE_bind (i.e., deviation of Δ*G*_bind_^pol^), and relative to the reference solvation (hydration) energies of small molecules, RMSE_solv (i.e., deviation of Δ*G*_pol_). As we noted in the Introduction, the main purpose of including the small molecules solvation energies into the binding energy parameters optimization is to try to reduce the overfitting observed for our previous best set of atomic radii.^54^ Specifically, we minimize
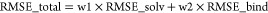
6where we explore several fixed values in the range from 0 to 1 for the relative weights of the solvation energies contribution, w1, and the binding energies contribution, w2. Thus, for example, the (w1:w2) combination with weights (0:1) corresponds to optimizing the radii against pure binding free energy references, while the opposite case (1:0) corresponds to the commonly employed radii optimization against solvation free energies of small molecules. As mentioned in the Introduction, both of these extremes are expected to be suboptimal; therefore, several in-between weights are tested here.

A set of small protein–ligand complexes is employed for the training and initial validation; see Materials and Methods. To make high-quality, deep optimization feasible, only the electrostatic (polar) parts of the total free energies are used as the reference targets.^54,69^

The most promising set of radii obtained at this stage is then fully tested directly against experimental binding free energies on a set of H–G complexes; see Materials and Methods. The main results presented below are for the optimization in the 5D parameter space^a^ of atomic radii for four key atom types, C, H, N, O, and the water probe radius, ρ_*w*_. The radius of the sulfur atom is not optimized and is set equal to its Bondi value, ρ_*S*_ = 1.80 Å.

The results of these optimizations are summarized in Table 2 for different combinations of relative weights (w1:w2) in the objective function for accuracies (RSMEs) of the solvation energies of small molecules and binding energies for protein–ligand complexes, respectively. Unless otherwise specified, the set of radii {ρ_W_, ρ_C_, ρ_H_, ρ_N_, and ρ_O_} corresponding to the lowest minimum of the objective function is shown, denoted by “OPT_BIND (w1:w2)”. Several nearby local minima (OPT2_BIND, OPT3_BIND, etc.) for the best found (w1:w2) weights combination are presented in the Supporting Information for completeness sake, and are discussed later, in the context of testing against experimental binding free energies.

The first clear conclusion that emerges is that the radii optimized exclusively against small molecule HFE [set OPT_SOLV (1:0), the last column in Table 2] perform relatively poorly in the context of accuracy of Δ*G*_bind_, with the corresponding RMSE_bind for protein–ligand complexes being several times higher than for all other explored (w1:w2) options, for both the Train and Validation sets. This result is consistent with previous observations for implicit^68,76^ and even some explicit water models.^12,108^ Our conclusion that optimizing the radii against small molecule aqueous solvation free energies is suboptimal for binding is particularly strong for two reasons. First, we are comparing the results of the same optimization algorithm, and second, a deep optimum has likely been reached^69,109^ in each (w1:w2) case, which accentuates the effect.

On the opposite end of the spectrum is the case (0:1)—radii optimized against Δ*G*_bind_ alone,^69^ leftmost column in Table 2. The value of RMSE_bind = 3.94 kcal/mol over the Train set is the lowest one compared to all other optima in this table because in this case, the objective function being minimized is RMSE_total = RMSE_bind. However, a comparison with the corresponding error for the Validation set, Table 2, reveals a noticeable increase in the RMSE_bind, to 6.62 kcal/mol, which is a clear indication of significant overfitting.

To combat the overfitting, we explore intermediate (w1:w2) cases, in which the accuracy of polar components of solvation free energies of small molecules is gradually “mixed into” the objective function. This is the crux of our strategy. Upon changing the relative contribution of RMSE_solv, w1, from 0 to 0.1, RMSE_bind goes up, from 3.94 to 4.60 kcal/mol for the Train set (15% increase), third column of Table 2. This slight decrease in the accuracy of the binding energies can be expected since we are no longer optimizing against pure RMSE_bind. Upon further increase of w1 from 0.1 to 0.3, the optimization algorithm reports the same optimal radii set since this small change in the relative weights, leading to a slightly different value of the objective function (RMSE_total) in the 5D parameter space, apparently does not change the position and relative ranking of the lowest identified minimum for the (0.1:1) optimization. The most important observation in moving from the (0:1) to (0.1:1) case is that the overfitting is now essentially gone, with the Train and Validation set RMSE_bind values being 4.60 and 4.99 kcal/mol, respectively. Also note that the increase in RMSE_bind for the Train set alone, 4.60 vs 3.94 kcal/mol, is not as extensive as the drop in the corresponding RMSE_bind for the Validation set, from 6.62 to 4.99 kcal/mol.

As the relative weight w1 of the small molecule solvation contribution becomes equal to that of the binding, case (1:1), the relative order and positions of the minima of the objective function change, leading to yet another set of optimal radii. The corresponding RMSE_bind increases relative to (0.1:1) and (0.3:1) cases, as well as the gap between the Validation and Train sets, suggesting a less predictive ability for binding energies of an optimal radii set resulting from optimization with such an “excessive” mixing.

In summary, based on the performance of Train and Validation sets, the best candidate for further testing is the radii set derived from optimization against a mix of small molecule HFEs and binding free energies, with relative weights of (0.1:1) and (0.3:1). Since increasing the relative weight of the small molecules contribution from 0.1 to 0.3 does not change this optimum and its accuracy, in what follows, we will focus on the (0.1:1) optimum and call the corresponding radii set OPT_BIND5D, omitting relative weights specification. It should be noted that while RMSE_solv makes a smaller contribution to RMSE_total compared to RMSE_bind due to its lower weight, the small molecule data set used for HFE calculation is an order of magnitude larger than the protein–ligand data set used for the free energy of binding calculations. This relative diversity of the HFE data set likely contributes to the reduced over-fitting. This benefit of adding RMSE_solv to the objective function should not be underestimated.

In Table 3, we list the radii corresponding to the OPT_BIND5D set, as well as the “pure binding” OPT_BIND (0:1) set (denoted as “OPT1” in ref.,^69^ where it was originally derived), along with the three common, well-established radii sets. For each radii set, the corresponding RMSE values for the polar components of the Δ*G*_bind_ and Δ*G*_solv_^s.m.^ energies for the Train and Validation sets are presented as well. One can see that both the OPT_BIND5D and the OPT_BIND (0:1) radii sets produce more accurate Δ*G*_bind_ values than BONDI^b^, PARSE, or ZAP-9^c^ radii sets for either the Train or Validation molecule sets. Also, our new OPT_BIND5D radii set, despite being optimized mostly (but not exclusively) for binding energies, still provides reasonably accurate values for the polar components of the solvation free energies of small molecules, the accuracy being not too different from that obtained with the widely used BONDI set and more accurate than using the PARSE set. Note that atomic radii sets optimized exclusively for binding (and showing good accuracy) are not guaranteed to perform nearly as well for HFEs of small molecules.^27^ In the case of H–G complexes, the accuracy loss was attributed^27^ to the need to compensate for excessively favorable net electrostatic interactions in the H–G systems.

A reasonably good accuracy of the new OPT_BIND5D radii set in predicting hydration energies of small molecules is another indication of its low overfitting as well as of the possibility that this set may generalize well outside of the Train set of molecules. The values of the optimal radii in the new radii sets, including OPT_BIND5D, should be treated as effective parameters that define the solute/solvent DB^57^ within the specific implicit solvent model considered in this work. Note that the DB itself is not an experimentally measurable quantity,^57^ even though it conforms to certain physical constraints.^69,110^ While the overall magnitudes of the effective atomic radii are not unreasonable, we refrain from attempting to overinterpret these specific values as real physical radii. One clear trend that emerges is that the radii sets optimized for binding are distinctly different from optimized against HFEs of small molecules; the regions of the 5D parameter space where these families of optimal solutions are found are substantially separated. The basic underlying reason is the inadequacy of the physical approximation in the relatively simple (but effective) implicit solvent models used in this work.

In what follows, we will explore performance of these two new radii sets [OPT_BIND (0:1) and OPT_BIND5D] as well as two common radii sets (mbondi and PARSE) directly against experimental binding free energies over a test set of H–G complexes. Note that these complexes are very different from those used so far in the training and validation, providing a useful and stringent accuracy test for the new radii.

### Testing the Accuracy of the New Optimal Radii Sets against Experiment Using H–G Systems

**Figure 3:**
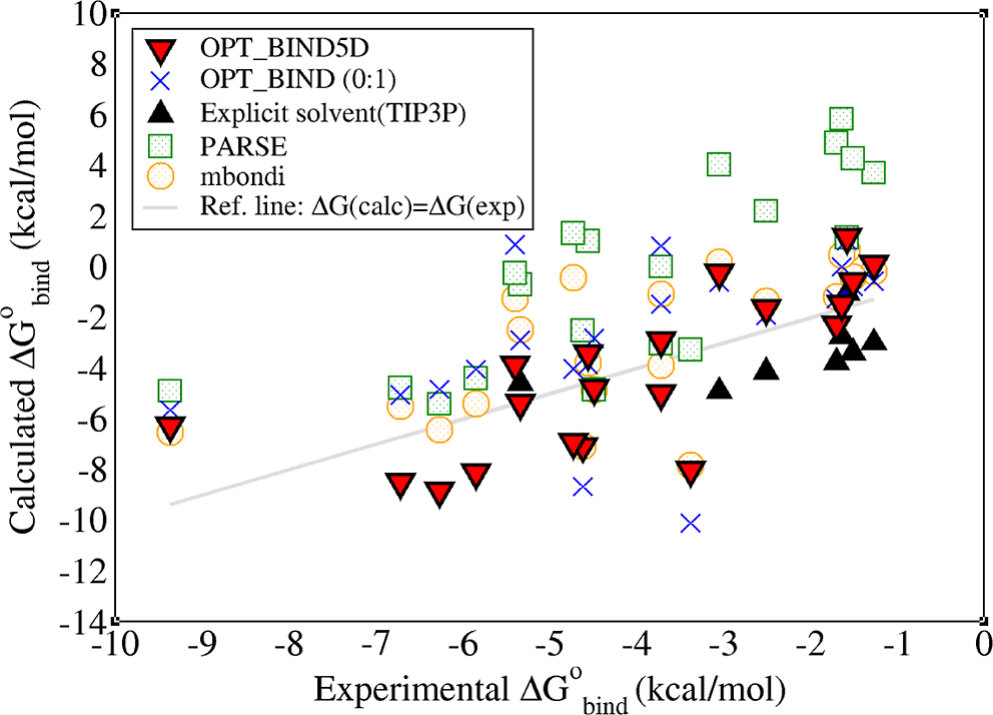
Estimates of Δ*G*_bind_^0^, calculated by the MM-GBSA method utilizing several radii sets, as well as the explicit solvent estimates (for low affinity complexes), are compared with the experimental binding free energies for 20 H–G systems at the 0.154 M monovalent salt concentration. The reference line represents perfect agreement between computed and experimental values.

The overall evaluation of the OPT_BIND5D radii set in enhancing the accuracy of MM-GBSA calculations for predicting Δ*G*_bind_^0^ across 20 H–G systems is shown in Figure 3 and Table 4. Notably, the OPT_BIND5D set outperforms other radii sets by achieving the lowest RMSE and MSE of binding energies relative to experimental values. Conversely, PARSE radii exhibit the largest RMSE and MSE, and the OPT_BIND (0:1) radii set shows the lowest Pearson’s (*r*) correlation, indicating their relatively poor accuracy in predicting Δ*G*_bind_ outside the training set.

In our analysis, we also evaluated the accuracy of the OPT2_BIND and OPT3_BIND radii sets [corresponding to 2-nd and 3-d local minima in the protein–ligand optimization with (0.1:1) relative weights, as compared to OPT_BIND5D (=OPT1_BIND), which is the lowest minimum] in predicting Δ*G*_bind_^0^ across the entire data set of H–G systems. These two sets exhibit inferior accuracy in terms of H–G binding RMSE relative to experiment, producing values of 2.33 and 2.37 kcal/mol, respectively. These RMSE values are larger than the value of the OPT_BIND5D set of 2.03 kcal/mol but still substantially better than the corresponding value of 2.96 kcal/mol for the OPT_BIND (0:1) radii set, optimized without small molecules hydration contribution.

This finding, which demonstrates the highest accuracy of OPT_BIND5D on H–G systems compared to the “next best” OPT2_BIND and the OPT3_BIND sets, is noteworthy. Mathematically, it means that the overall structure of the basin around the main OPT_BIND5D minimum is preserved, even though the model was trained on a fundamentally different category of complexes (protein–ligand), and only against the electrostatic (polar) components of the Δ*G*_bind_ and Δ*G*_solv_^s.m.^ energies estimated using TIP3P explicit water model. In turn, this robustness of the main optimum lends further support to our computational procedure and is a strong indication of transferability of the OPT_BIND5D set. This observation lends additional credence to our proposed optimization strategy and to the role of OPT_BIND5D as a good candidate optimal radii set for MM-GBSA estimates.

A direct comparison with other solvent models, such as the most widely used, but computationally much more demanding, explicit water models, is not straightforward as the resulting accuracy depends not only on the quality of the solvent model but also on the specific method used to estimate Δ*G*_bind_^0^. Still, it is useful to place the accuracy of OPT_BIND5D in the context of what one might expect in practice compared to using general-purpose explicit solvent models. Specifically, the accuracies of TIP3P,^111^ OPC,^112^ and TIP4P-Ew^113^ in predicting experimental Δ*G*_bind_^0^ were evaluated^12^ on a set of H–G complexes similar to those used in this work, with the reported RMSE values of 1.7, 2.0, and 2.9 kcal/mol, respectively. This comparison suggests that MM-GBSA estimates based on the radii set of the OPT_BIND5D model (Table 4) are likely competitive with those based on common fixed charge explicit water models, at least in the context of H–G binding. A more direct comparison with TIP3P will be presented below.

### Detailed Analysis by H–G Subgroups

To provide a more detailed evaluation of each radii set listed in Table 4, we divided the entire H–G data set into three subgroups based on the complex charge and affinity characteristics of the systems: “highly charged”, “near neutral”, and “low affinity”. For each of these H–G subgroups, RMSE, MSE, MAE, SRC, and r-correlation values against experiment are presented in Table 6. Notably, our new OPT_BIND5D set consistently demonstrates the lowest RMSE within each subgroup, underscoring its accuracy in predicting Δ*G*_bind_^0^ across various types of systems. On the other hand, PARSE radii exhibit the weakest accuracy in all subgroups. One intriguing subgroup of the H–G complexes is the “low affinity” category. This subgroup primarily comprises the H–G systems with low experimental Δ*G*_bind_^0^ values, see Table 5 in the Supporting Information and Figure 3 for the weak binders. Analyzing the configurations of the H–G complexes in this subgroup using the snapshots from the corresponding MD simulations in the explicit solvent, we found that in many instances, the guest molecules temporarily exit the host pockets. These “unbound” configurations were identified by using a system-dependent dynamic threshold and are further analyzed. What sets this subgroup apart is that it is the only one that provides an opportunity to compare the Δ*G*_bind_^0^ values obtained from MM-GBSA calculations with those directly computed using the binding–unbinding statistics in the explicit solvent (TIP3P) simulations (eq 1 without further approximations that often accompany estimates of Δ*G*_bind_ in the explicit solvent). Employing OPT_BIND5D radii in the MM-GBSA calculations of Δ*G*_bind_^0^, along with considering proper salt concentration effect and the entropy offset, we achieve the RMSE of 1.55 kcal/mol, MSE of 1.03 kcal/mol, MAE of 1.19 kcal/mol, and correlation of *r* = 0.89 relative to experiment for this “low affinity” subgroup. The above accuracy is on par with the accuracy of the explicit solvent (TIP3P) estimates with the RMSE of 1.67 kcal/mol, MSE of −1.25 kcal/mol, MAE of 1.57 kcal/mol, and correlation of *r* = 0.66 relative to experiment^d^. The above comparison is arguably closest possible to a direct comparison between two very different solvent models: no approximations are used to estimate Δ*G*_bind_^0^ directly from the statistics of binding/unbinding events in the explicit solvent (and, of course, the set of test structures, the underlying gas-phase force-field, including partial charge, are exactly the same). While the fixed-charge, 3-point TIP3P model is certainly not the most advanced water model available,^29^ and the implicit solvent model can only be expected to perform as well as the explicit model it was parameterized against, it appears that low-affinity H–G complexes may present a challenge to even significantly more sophisticated, polarizable water models.^25^

Finally, we verified the robustness of our direct, binding–unbinding statistics based estimates of Δ*G*_bind_^0^ using eq 1 by conducting longer (3 μs) MD simulations using OPT_BIND5D, on β-CD-6-p—a system with the smallest experimental Δ*G*_bind_^0^ value in our data set, which exhibits 25 binding/unbinding events (Figure 2). From the final results, given in Table 5, it is clear that the explicit solvent results are robust to changing the trajectory length and its temporal resolution.

Promising results with OPT_BIND5D radii in enhancing MM-GBSA calculations and achieving high accuracy in Δ*G*_bind_^0^ predictions suggest that this new set of atomic radii could be considered as the default choice in MM-GBSA setups in the near future.

### Notes on the Salt Dependence of Δ*G*_bind_^0^

Understanding the mechanisms of protein–protein binding necessitates a comprehensive model of biological macromolecular interactions within ion-rich environments. At the forefront of this investigation is the role of electrostatic forces, which substantially influence the sensitivity of Δ*G*_bind_^0^ to alterations in salt concentrations. Therefore, calculating the variations in the electrostatic component at different ionic strengths allows to estimate the sensitivity of protein–protein interactions to the concentration of mobile ions.^19,114−125^

**Figure 4:**
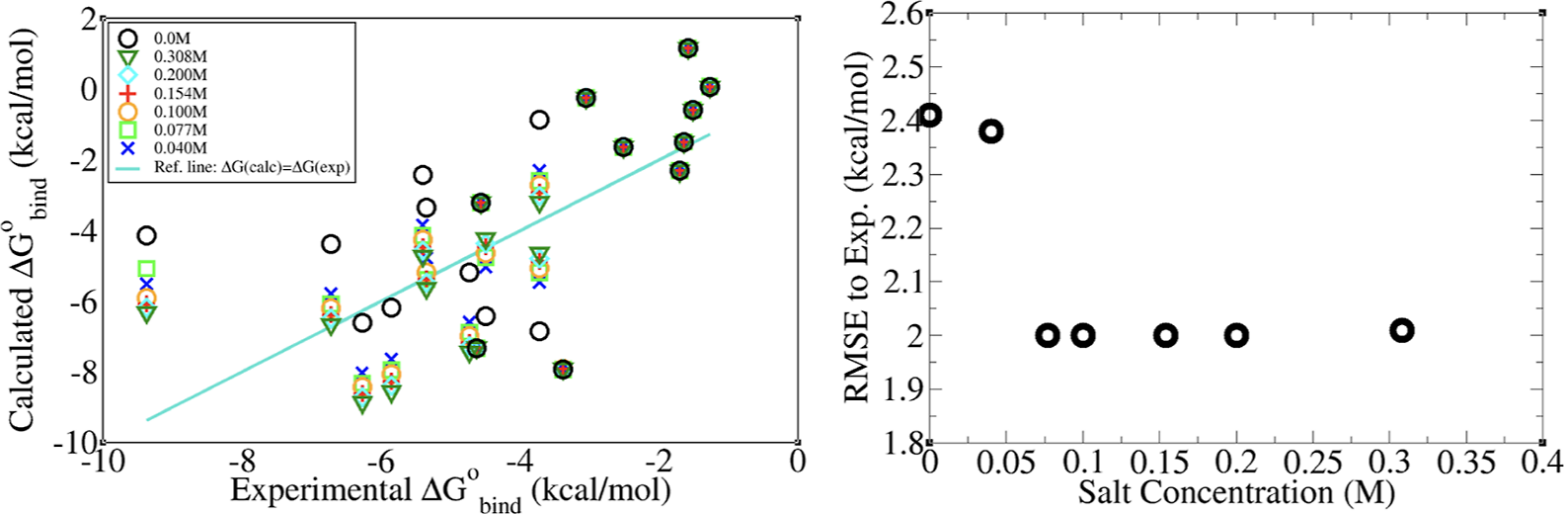
Left panel: The effect of varying monovalent salt concentration from 0 to 0.3 M on the implicit solvent estimates of Δ*G*_bind_^0^ is negligible for the low-affinity complexes but can be pronounced for the high-affinity ones. In all cases, the change in computed Δ*G*_bind_^0^ estimates is minimal when the salt concentration fluctuates by ∼50% around the physiological level. Right panel: The RMSE values for the MM-GBSA estimates relative to the experimental Δ*G*_bind_^0^ for 20 H–G complexes at different monovalent salt concentrations show best agreement with experiment over a range of concentrations around the physiologically relevant value of 0.154 M.

In line with previous explicit solvent studies,^19^ our observation emphasizes a strong dependence of the implicit solvent (MM-GBSA) Δ*G*_bind_^0^ estimates on the salt concentration. Namely, increasing the monovalent salt concentration from 0 (AMBER default value) to 0.154 M (physiological) consistently improves all MM-GBSA estimates reported here, regardless of the radii set employed (Figure 4) (for reference, the individual Δ*G*_bind_^0^ estimates, computed at 0 and 0.154 M monovalent salt concentrations, are given in the Supporting Information). Our findings indicate that incorporating approximate salt effects in MM-GBSA yields satisfactory results: the highest correlation with experimental data is observed near the physiological salt concentration where experimental Δ*G*_bind_^0^ were recorded (Figure 4, left). The computed binding free energy of H–G systems demonstrates minimal sensitivity to the variations in the salt concentration within a range from approximately 50% above to 50% below the physiological level (Figure 4, right). Still, correct setting of the salt concentration is critical for the best accuracy. This is particularly important for complexes with high affinity (Δ*G*_bind_^0^ less than −3 kcal/mol) as utilizing 0 salt concentration in computational studies of such complexes can lead to significant inaccuracies (Figure 4, left). In our data set of H–G complexes, the majority of the highly charged complexes belong to the “high affinity” subset, which might suggest a correlation between the magnitude of the salt effect and the charge of the H–G complex. However, a previous study^125^ on a much larger and more diverse data set indicates that the magnitude of the salt effect is not necessarily correlated with the charge of the complex; rather, it is governed by a complex interplay between the shape and charge distribution of the molecules. A previous study^25^ on the effect of salt concentration on predicting H–G binding free energies using polarizable water models assessed the impact at three different NaCl concentrations: 0, 200, and 1000 mM. The results indicated that ion concentration significantly influences the free energy of binding, with stronger binding observed at higher salt concentrations. This finding is consistent with another experimental study,^126^ which demonstrated that increasing salt concentrations enhances binding for OA cavitands. Our research extends these observations across a broader range of salt concentrations and shows that using the correct experimental salt concentration in the implicit solvent estimates of Δ*G*_bind_^0^ results in the best agreement with experiment.

## Conclusions

In this work we have proposed, and thoroughly evaluated, a novel optimization procedure for developing key parameters (atomic and water probe radii) for estimates of ligand binding energetics in the implicit solvent. The outcome is a set of four atomic radii for H, C, N, and O atoms and one for the water probe, which is a 5D parameter set that we call the OPT_BIND5D.

The proposed optimization procedure has several unique features. It attempts to minimize the combined deviation from reference for both binding free energies of a set of small protein–ligand complexes and a set of hydration free energies (HFEs) of small molecules. The computationally demanding optimization becomes feasible through the use of the numerical GB (GBNSR6) flavor used for on-the-fly evaluations of the electrostatic components of the HFEs and binding free energies. The procedure attempts to find a strong optimum in the 5D parameter space, which becomes possible through the use of the massively parallel implementation of the VTDIRECT95 deterministic optimization method.

Our first methodological conclusion is that using a small, but still non-negligible (10–30%), contribution of the small molecule HFE component in the objective function reduces overfitting to the necessary small training set of protein–ligand complexes, while the accuracy with respect to the HFE reference remains acceptable, comparable to that of several common radii sets with the same number of parameters. This observation suggests that the resulting OPT_BIND5D radii are likely to generalize outside the set of neutral small protein–ligand complexes used for the radii optimization, an assumption that we have thoroughly tested next. Namely, we have presented a comprehensive evaluation of the accuracy enhancement achieved through the use of the newly developed set of atomic radii, OPT_BIND5D, in MM-GBSA calculations on H–G systems. This test set encompasses a broad range of experimental Δ*G*_bind_^0^ values and different charge states across 20 H–G systems from the two benchmark data sets. These systems represent a close to ideal test case for this work, and not only because they differ substantially from the structures used in the training. We, as well as others,^12^ argue that these small, relatively rigid systems are more suitable than typical protein–(small molecule) or protein–protein complexes for the goal of isolating and improving the effects of solvent accuracy on binding free energy estimates. In particular, microsecond-long trajectories used in this work appear to be long enough to deliver adequate sampling of the conformational space of these small structures, resulting in the reasonably converged Δ*G*_bind_ estimates.

We have compared the Δ*G*_bind_^0^ predictions for H–G systems obtained with our OPT_BIND5D radii against those from three atomic radii sets: two well-established sets mbondi and PARSE and a set (OPT_BIND (0:1)) optimized against binding free energies only, without a hydration free energy of small molecules component present during the optimization OPT_BIND5D. We find that OPT_BIND5D exhibits the lowest RMSE of 2.03 kcal/mol, the lowest MSE of −0.13 kcal/mol, the lowest MAE of 1.68 kcal/mol, and the high correlation of *r* = 0.79 with experimental Δ*G*_bind_^0^ values. The SRC value provides evidence supporting the higher accuracy of OPT_BIND5D in predicting Δ*G*_bind_^0^. This superior performance of the OPT_BIND5D radii set in predicting Δ*G*_bind_^0^ relative to that of other radii sets tested here is seen separately for different subsets of H–G systems with various charge states and binding affinities. The OPT_BIND5D set also demonstrates a reasonable performance in predicting HFEs of small molecules, at the level of BONDI radii, and significantly better than the performance of PARSE radii. This observation suggests that OPT_BIND5D may be considered as a general radii set candidate suitable for other GB-based implicit solvent computations, not just those focused on estimates of binding energies.

A direct comparison of the accuracy of OPT_BIND5D accuracy with that of the explicit solvent models is not straightforward as the predictions depend not only on the quality of the solvent model but also on the specific method used to estimate Δ*G*_bind_^0^. With this caveat, the accuracy of MM-GBSA estimates utilizing the OPT_BIND5D set on H–G systems appears to fall within the range seen previously for common general-purpose water models such as TIP3P, OPC, and TIP4P-Ew, evaluated on a set of similar, though not identical, H–G complexes. For low-affinity complexes used in this work, a much more direct comparison with TIP3P-based estimates was possible by evaluating Δ*G*_bind_^0^ directly, i.e., without further approximation, through statistics of binding/unbinding events observed in long enough MD simulations in TIP3P on the same set of H–G structures. This comparison revealed that the proposed OPT_BIND5D used in MM-GBSA is just as accurate (RMSE = 1.55 kcal/mol, MSE = 1.03 kcal/mol, MAE = 1.19 kcal/mol *r* = 0.89) as the direct estimate in TIP3P (RMSE = 1.67 kcal/mol, MSE = −1.25 kcal/mol, MAE = 1.57 kcal/mol, *r* = 0.66), with the caveat that the test subset of low-affinity structures was relatively small. These findings indicate the promise of the new radii set in computational estimates of ligand binding energetics while providing yet another rationale for the use of H–G systems for the testing.

Additionally, this study highlights the importance of salt effects in MM-GBSA estimates of binding free energies: using the experimental salt concentration for implicit solvent estimates of Δ*G*_bind_^0^ achieves the best agreement with the experimental results.

The work has several limitations. First, we have tested the new radii against experimental binding affinities on a set of H–G complexes only. A future thorough testing will eventually determine how well the new radii will perform in estimates of binding free energies for complexes involving macromolecules, including protein–(small molecule), protein–(nucleic acid), and (nucleic acid)–(small molecule) complexes. In this respect, it is encouraging that radii that perform well in the context of GB-based estimates of binding free energies of H–G systems were shown recently to also deliver comparable accuracy of protein–ligand binding affinities.^27^ We hope that the results presented here are encouraging enough for practical use and further testing, which will ultimately reveal how well the new set is. Second, the end-point single-trajectory MM-GBSA method used to estimate the free energy of binding has its own limitations: in particular, the benefits of “noise cancellation” are offset, to a degree, by the inevitable neglect of possible conformational differences between the same structure in the bound and unbound state. We note that despite these limitations, we do see a clear accuracy improvement with the new optimized radii set, which we attribute to the improvement in the accuracy of solvent description. Third, even though the novel optimization pipeline for developing atomic radii for binding calculations has yielded a strong optimum, residual errors larger than *kT* remain. We may have reached the limit of what the two-dielectric linear response continuum solvent model can deliver, and we attribute the remaining errors to the inherent limitations of the underlying continuum electrostatic methodology used in the GB models^e^. A hierarchy of approximations separates the GB/PB approach from the more accurate explicit solvent models, and, ultimately, from reality.^87,127^ For example, the GB model, as well as the PB model for that matter, takes into account only the dipole polarization of solvent; the models do not explicitly account for charge hydration asymmetry^128^ or other higher water multipole effects^129^ present in real water. These limitations highlight the necessity to consider more sophisticated “beyond GB/PB” implicit solvent models for protein–ligand binding estimates, such as implicit water multipoles GB model,^110^ 3D-RISM models,^130−133^ integral equation formalism,^134^ explicit/implicit hybrid solvent models,^135,136^ or models based on the fundamental variational principle.^137−144^ The challenge is to find an acceptable compromise between accuracy and efficiency.

To conclude, our study introduces a promising radii set, namely, OPT_BIND5D, worth considering for routine MM-GBSA calculations. This set improves the accuracy of computationally relatively inexpensive binding free energy predictions in the implicit solvent, which is crucial for enhancing the effectiveness of computer-aided drug design.

## Data Availability

Additional data that support the findings of this study are available in the Supporting Information, or from the corresponding author upon reasonable request. A set of scripts to set up OPTBIND5 radii for MM-GBSA calculations, along with the AMBER topology and coordinate input files for all of the H-G systems used in this study, are available at https://github.com/Onufriev-Lab/OPTBIND5D. AmberTools set of programs, which includes the MMPBSA module, is available at https://ambermd.org/.

## Supporting Information Available

Details of the additional minima of the optimized radii sets in 5D, comparisons of the corresponding train RMSE values for solvation and binding energies, optimization attempts in the larger parameter space, from 6D to 10D, and all of the free energy components of the computed Δ*G*_bind_^0^ values for H–G complexes for all of the tested radii sets, at both 0 and 0.154 M monovalent salt concentrations (PDF)

## Footnotes

a An exploration of higher-dimensional radii space, up to 10D, is presented in the Supporting Information. No clear evidence of noteworthy improvements compared to the 5D case is observed; in this work, we have not pursued further exploration and testing of these higher-dimensional radii sets.

b A version of Bondi atomic radii used in AMBER.

c For consistency with the previous work on radii optimization for binding in the 5D parameter space, we use the same subset of ZAP-9 radii as in ref (69).

d Some beneficial cancellation of error between various terms of the implicit solvent model, which add up to the final Δ*G*_bind_, can never be excluded. But the fact that the accuracy of the new model is essentially that of TIP3P lends an additional support to self-consistency of the optimization procedure, which used TIP3P as the reference.

e Note that the GB model used here is GBNSR6, which approximates the numerical PB closely, essentially without adjustable parameters. More approximate GB flavors may need their parameters reoptimized for optimal performance with the proposed radii set.
