# Supplementary material for: Optimal Dielectric Boundary for Binding Free Energy Estimates in the Implicit Solvent
Source: J Chem Inf Model. 2024 Dec 10;64(24):9433–48. doi: 10.1021/acs.jcim.4c01190 (PMC11684022; doi:10.1021/acs.jcim.4c01190)
Supplement: Supplementary file 1 — ci4c01190_si_001.pdf [file ci4c01190_si_001.pdf]

Optimal Dielectric Boundary for Binding Free Energy Estimates in the Implicit Solvent

Negin Forouzesh,[†,⊥] Fatemeh Ghafouri,[‡,⊥] Igor S. Tolokh,[¶] and Alexey V. Onufriev[*,¶,§,‖]

†*Department of Computer Science, California State University, Los Angeles, CA 90032, USA*

‡*Genetics Bioinformatics and Computational Biology, Virginia Polytechnic Institute & State University, Blacksburg, VA 24061, USA*

¶*Department of Computer Science, Virginia Polytechnic Institute & State University, Blacksburg, VA 24061, USA*

§*Department of Physics, Virginia Polytechnic Institute & State University, Blacksburg, VA 24061, USA*

‖*Center for Soft Matter and Biological Physics, Virginia Polytechnic Institute & State University, Blacksburg, VA 24061, USA*

⊥*These authors contributed equally to this work*

E-mail: alexey@cs.vt.edu

Supporting Information Available

5D Optimized radii: relative ranks 1-5, (0.1:1) relative weights case

We also evaluated the accuracy of the OPT2_BIND and OPT3_BIND radii sets in predicting experimental $\Delta G^0_{\mathrm{bind}}$ across the entire dataset of the H-G systems used in this work. These

Table 1: Ranked optimized radii sets in 5D parameter space (radii values in Å) and corresponding Train RMSE values (in kcal/mol, with (0.1:1) relative weights of RMSEs for polar components of binding energies and small molecules solvation energies). Radius of sulfur atom is not optimized (*) and set equal to its Bondi value.

Radii Set	OPT_BIND5D	OPT2_BIND	OPT3_BIND	OPT4_BIND	OPT5_BIND
ρ_W	1.35	1.38	1.21	1.37	1.06
ρ_C	2.23	2.23	2.26	2.17	1.35
ρ_H	1.47	1.55	1.40	1.49	1.74
ρ_N	2.37	1.85	1.73	1.16	2.48
ρ_O	1.09	1.08	1.14	1.16	1.17
ρ_S	1.80*	1.80*	1.80*	1.80*	1.80*
Train set					
RMSE_solv	2.14	1.89	1.76	1.93	2.65
RMSE_bind	4.60	4.76	5.01	5.10	5.07
RMSE_total	4.81	4.94	5.19	5.29	5.34

additional optima, Table 1, correspond to the 2nd and 3rd local minima of the same optimization described in the main text. (Note that OPT_BIND5D would be OPT1_BIND in this notation.) The optimization targeted the polar components of the free energies of small molecule hydration and of protein-ligand binding, with (0.1:1) relative weights of the corresponding RMSE values. We find that these two sub-optimal sets exhibit inferior accuracy (compared to OPT_BIND5D set) in terms of H-G binding RMSE relative to experiment, the corresponding values are 2.33 kcal/mol and 2.37 kcal/mol, respectively. The corresponding OPT_BIND5D set performance (RMSE = 2.03 kcal/mol) is presented in Table 4 of the main text.

Following an idea explored in Ref.,[1] we attempted to enhance the accuracy through averaging over the ΔG^0_{bind} values derived from these three distinct OPT_BIND radii sets, Eq. 1:

$$\Delta G^0_{\text{average}} = \frac{1}{3}\left(\Delta G^0_{\text{OPT\_BIND5D}} + \Delta G^0_{\text{OPT2\_BIND}} + \Delta G^0_{\text{OPT3\_BIND}}\right); \tag{1}$$

the resulting RMSE of $\Delta G^0_{\text{average}}$ is 2.14 kcal/mol relative to experiment, which is larger than the error due to the use of OPT_BIND5D alone.

One plausible explanation for the lack of expected accuracy improvement is that OPT2_BIND and OPT3_BIND radii sets are not “different enough” from OPT_BIND5D to reduce the residual systematic errors, and hence to provide accuracy benefits of the averaging approach. Note that in Ref.[1] the averaging included the Bondi set.

6D Optimized radii: ranks 1-5, (0.1:1) relative weights case

Table 2: Ranked optimized radii sets in 6D parameter space (radii values in Å) and the corresponding Train set RMSE values (in kcal/mol).

Radii Set	OPT1_BIND	OPT2_BIND	OPT3_BIND	OPT4_BIND	OPT5_BIND
ρ_W	1.38	1.37	1.37	1.37	1.06
ρ_C	2.23	2.25	2.23	2.23	1.77
ρ_H	1.50	1.49	1.60	1.49	1.50
ρ_N	2.36	1.87	1.28	2.34	2.12
ρ_O	1.09	1.08	1.04	1.10	1.23
ρ_S	2.81	2.83	2.81	2.33	3.07
Train set					
RMSE_solv	2.13	1.89	1.90	2.13	2.36
RMSE_bind	4.28	4.35	4.67	4.73	5.15
RMSE_total	4.50	4.53	4.86	4.94	5.39

Optimization in larger parameter space

In the main text we have explored a "minimal" set of distinct radii, *i.e.,* sets with single radius value per chemical element (4 main elements and water probe have been considered), leading to 5-dimensional (5D) parameter space, *e.g.,* Table 1, (or 6-dimensional (6D) space if the radius of sulfur atom (ρ_S) was also optimized, Table 2). Here we explore whether further extending the parameter space may lead to more accurate ΔG_{bind}. We use the same Train and Validation sets of molecules and the same objective function (RMSE_total) with (0.1:1) relative weights of RMSE_solv and RMSE_bind, as described in the main text.

It is well known that the position of the dielectric boundary depends to some extend on the strength of the local electric field,[2–4] which suggests making atomic radii explicitly dependent on the partial atomic charges of the corresponding atoms. In what is arguably the simplest version of the approach, we assign two distinct atom sub-types per element, a "low charge" and a "high charge" ones, and have their radii optimized as separate parameters. The assignment is based on an intuitive partitioning of the distributions of atomic partial charges q found in the corresponding pqr-files. These distributions are approximately bi-

modal, which we partition as follows:

ρ_{C^+} if $q_C \geq +0.25e$, ρ_{C^-} if $q_C < +0.25e$;

ρ_{H^+} if $q_H \geq +0.20e$, ρ_{H^-} if $q_H < +0.20e$;

ρ_{N^+} if $q_N \geq -0.70e$, ρ_{N^-} if $q_N < -0.70e$;

ρ_{O^+} if $q_O \geq -0.75e$, ρ_{O^-} if $q_O < -0.75e$,

where the "+" sign indicates the "high charge" sub-type, and "-" indicates the "low charge" sub-type.

Table 3: Optimized sets of water probe and atomic radii (Å) (with (0.1:1) relative weights of RMSE_solv and RMSE_bind in the objective function) and corresponding Train/Validation RMSE values (kcal/mol) in 5D, 6D (with sulfur optimization) and larger parameter spaces. (*) denotes not optimized Bondi radius value.

Radii Set	OPT_BIND5D (5D)	OPT1_BIND (6D)	OPT1_BIND (8D)	OPT1_BIND (10D)
ρ_W	1.35	1.38	1.37	0.90
ρ_{C^+}	2.23	2.23	2.11	1.61
ρ_{C^-}	2.23	2.23	2.11	1.39
ρ_{H^+}	1.47	1.50	1.57	0.75
ρ_{H^-}	1.47	1.50	1.63	1.47
ρ_{N^+}	2.37	2.36	1.99	1.60
ρ_{N^-}	2.37	2.36	1.99	2.85
ρ_{O^+}	1.09	1.09	1.10	1.59
ρ_{O^-}	1.09	1.09	1.25	1.56
ρ_S	1.80*	2.81	2.81	2.82
Train set				
RMSE_solv	2.14	2.13	2.14	2.85
RMSE_bind	4.60	4.28	4.31	4.48
RMSE_total	4.81	4.50	4.53	4.77
Validation set				
RMSE_solv	2.48	2.38	2.37	2.76
RMSE_bind	4.99	5.04	5.18	5.13
RMSE_total	5.24	5.28	5.42	5.41

Table 3 shows the results of the previous optimizations in the 5D parameter space, new parameter optimizations in the 6D (added sulfur atom radius, ρ_S) and optimizations in higher parameter spaces (8D – with added hydrogen and oxygen atoms sub-types, and 10D – with additional nitrogen and carbon atoms sub-types).

Exploring these higher dimensions of the parameter space we find that the RMSE_total (with the same (0.1:1) weights in the objective function for all presented cases) decreases from 4.81 kcal/mol in 5D space to 4.50 kcal/mol in 6D space (optimization of S atom radius added). This reduction of the RMSE_total is almost exclusively due to the reduction of RMSE_bind, since the small molecules set for RMSE_solv does not have S atoms and is much less affected by this optimization. Since the H-G systems investigated in the main text do not contain sulfur atoms, we do not pursue further analysis of the 6D set in this work, but note that it is a promising candidate for a more detailed future investigation. However, going from 6D to 8D parameter space (two additional H and O atomic radii sub-types added), RMSE_total as well as RMSE_bind are not reduced. Then, going to 10D parameter space (additional C and N radii sub-types added), RMSE_total for the Train set of molecules slightly increases to 4.77 kcal/mol. These observations likely indicate the "curse of high dimension" and, consequently, the undersampling of the feasible parameter space. Table 3 also compares the objective function values when the new optimized radii (OPT_BIND5D) and the radii sets for higher dimensions (OPT1_BIND 6D, OPT1_BIND 8D, and OPT1_BIND 10D) are validated. The RMSE_total values indicate that OPT_BIND5D set leads to the most accurate estimation of solvation and binding free energies combined. Based on this finding, we have selected OPT_BIND5D set as the most promising candidate to be thoroughly tested against experiment (see the main text).

The three subgroups of Host-Guest complexes.

Table 4: Members of each H-G subgroup. In all H-G complexes the guest is always neutral, making the charge of the complex (Q) equal that one of the host. "Low affinity" complexes mostly belong to "Near neutral" subgroup.

Highly charged ($-9e \leq Q \leq -7e$)	Near neutral ($-1e \leq Q \leq +1e$)	Low affinity complexes
OA-6(G4)	α-CD-8-p	OA-4(G6)
OA-4(G6)	β-CD-9-p	β-CD-9-p
OA-4	β-CD-6-p	β-CD-6-p
OA-8(G5)	α-CD-6′-p	α-CD-6′-p
OA-3(G1)	α-CD-1-p	α-CD-1-p
OA-5(G2)	α-CD-7-p	
OA-7(G3)	β-CD-8-p	β-CD-8-p
OA-1	β-CD-5-p	
OA-2	α-CD-5-p	α-CD-5-p
OA-3	β-CD-3-p	β-CD-3-p

New OPT_BIND5D radii tested for accuracy of $\Delta G^0_{\mathbf{bind}}$ estimates against experiment: Host-Guest systems

Table 5: Comparison of experimental (second column) and computed $\Delta G^0_{\mathrm{bind}}$ values (kcal/mol) estimated using different radii sets and 0.154 M (NaCl) salt concentration.

H-G systems	Exp.	`mbondi`	PARSE	OPT_BIND (0:1)	OPT_BIND5D	Explicit (TIP3P)
OA-6(G4)	-9.37	-6.52	-4.89	-5.64	-6.26	N/A
OA-4(G6)	-5.34	-2.47	-0.70	-2.89	-5.37	-4.60
OA-4	-6.72	-5.50	-4.77	-5.05	-8.49	N/A
α-CD-8-p	-4.62	-7.07	-2.52	-8.67	-7.08	N/A
β-CD-5-p	-4.56	-3.78	1.02	-3.88	-3.43	N/A
OA-8(G5)	-3.72	-3.88	-3.05	-1.48	-5.00	N/A
α-CD-5-p	-2.51	-1.34	2.20	-1.91	-1.63	-4.14
β-CD-9-p	-1.7	-1.19	4.90	-1.26	-2.30	-3.78
β-CD-6-p	-1.27	-0.19	3.70	-0.56	0.09	-2.97
OA-3(G1)	-5.4	-1.23	-0.26	0.88	-3.87	N/A
OA-5(G2)	-4.73	-0.41	1.32	-4.02	-6.92	N/A
OA-7(G3)	-4.49	-4.86	-4.84	-2.82	-4.80	N/A
OA-1	-3.72	-1.06	0.01	0.82	-2.91	N/A
OA-2	-5.85	-5.39	-4.40	-4.03	-8.12	N/A
OA-3	-6.27	-6.43	-5.41	-4.83	-8.85	N/A
α-CD-6′-p	-1.51	-0.38	4.27	-0.78	-0.57	-3.39
α-CD-1-p	-1.58	0.67	1.15	1.04	1.17	-1.03
α-CD-7-p	-3.38	-7.83	-3.26	-10.11	-8.01	N/A
β-CD-8-p	-1.64	0.47	5.83	0.01	-1.47	-3.76
β-CD-3-p	-3.05	0.19	4.04	-0.61	-0.25	-4.92

Table 6: Comparison of estimated ΔG^0_{bind} values (kcal/mol) computed at 0.0 M salt concentration using different radii sets.

H-G systems	`mbondi`	PARSE	OPT_BIND (0:1)	OPT_BIND5D
OA-6(G4)	-4.80	-3.17	-3.92	-4.14
OA-4(G6)	-0.47	1.30	-0.86	-3.37
OA-4	-3.48	-2.75	-3.09	-6.47
α-CD-8-p	-6.93	-2.38	-8.53	-7.32
β-CD-5-p	-3.58	1.22	-3.68	-3.21
OA-8(G5)	-5.81	-4.98	-3.42	-6.84
α-CD-5-p	-1.34	2.20	-1.91	-1.64
β-CD-9-p	-1.21	4.88	-1.28	-2.31
β-CD-6-p	-0.21	3.68	-0.58	0.07
OA-3(G1)	0.18	-2.96	2.29	-2.42
OA-5(G2)	1.14	2.87	-2.47	-5.19
OA-7(G3)	-6.14	-6.11	-4.11	-6.41
OA-1	0.92	1.99	2.79	-0.87
OA-2	-5.22	-4.23	-3.86	-6.17
OA-3	-3.97	-3.28	-2.71	-6.61
α-CD-6′-p	-0.39	4.25	-0.8	-0.59
α-CD-1-p	0.66	1.15	1.03	1.16
α-CD-7-p	-7.66	-3.09	-9.94	-7.92
β-CD-8-p	0.44	5.80	-0.02	-1.49
β-CD-3-p	0.19	4.04	-0.61	-0.25

Table 7: Accuracy of the ΔG^0_{bind} estimates against experiment, computed for different subgroups in the H-G test set using different radii sets. The estimates are calculated for 0.0 M salt concentration. RMSE values, Mean Signed Error (MSE), and Mean Absolute Error (MAE) values are in kcal/mol.

	**All complexes**	Highly charged	Near neutral	Low affinity
		`mbondi`		
RMSE	3.19	3.94	2.20	2.44
r correlation	0.45	0.30	0.84	0.17
MSE	1.69	2.80	0.58	2.03
MAE	2.72	3.54	1.89	2.03
SRC	0.35	0.06	0.70	0.35
Bootstrap SRC	0.44	-0.05	0.77	0.37
		PARSE		
RMSE	4.93	4.51	5.24	5.92
r correlation	0.68	0.23	0.76	0.56
MSE	4.09	3.33	4.76	1.70
MAE	4.38	3.90	4.75	5.73
SRC	0.63	0.05	0.52	0.16
Bootstrap SRC	0.61	0.04	0.35	0.21
		OPT_BIND (0:1)		
RMSE	3.63	4.32	2.76	2.15
r correlation	0.29	0.40	0.81	0.28
MSE	1.79	3.63	-0.05	1.70
MAE	2.83	3.63	2.04	1.69
SRC	0.44	0.27	0.76	0.50
Bootstrap SRC	0.48	0.41	0.73	0.45
		**OPT_BIND5D**		
RMSE	2.36	2.49	2.21	1.70
r correlation	0.60	0.11	0.79	0.72
MSE	0.47	0.71	0.23	1.27
MAE	1.87	1.94	1.80	1.69
SRC	0.63	0.09	0.78	0.50
Bootstrap SRC	0.67	0.04	0.77	0.47

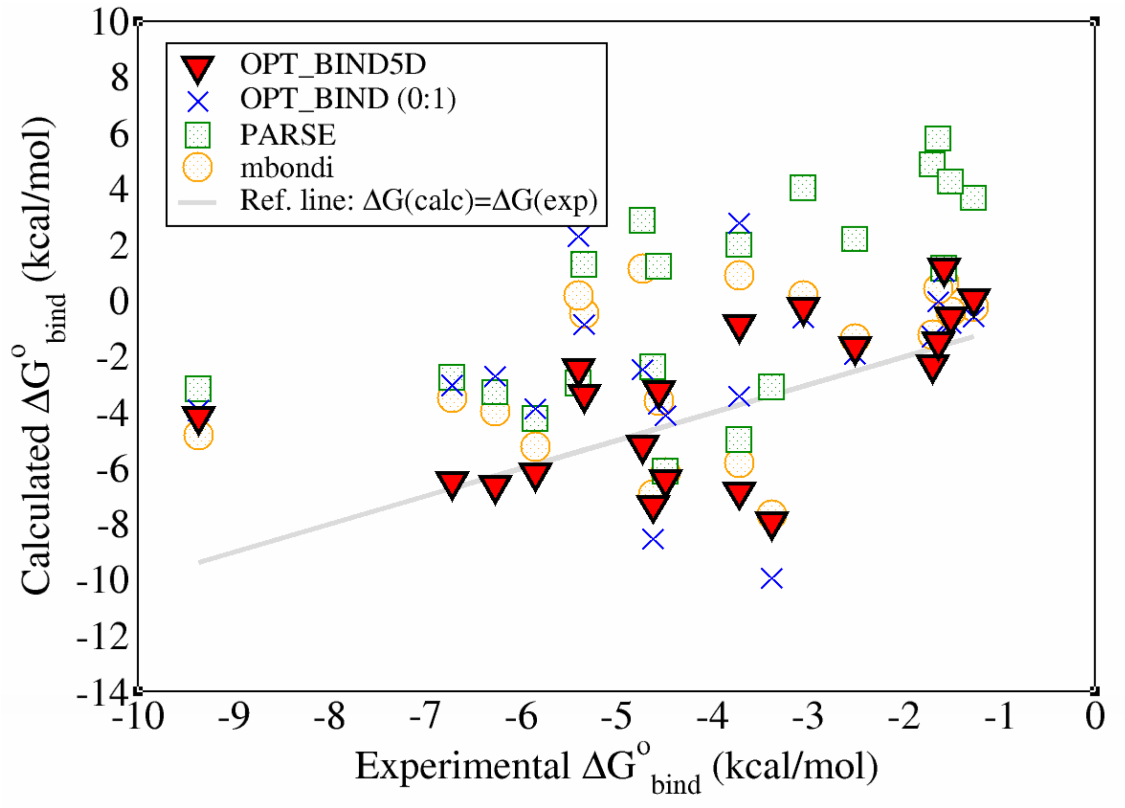

Figure 1: Accuracy of computational estimates of MMGBSA for ΔG^0_{bind} (in kcal/mol) employing various radii sets (with 0.00 M salt concentration) against the experimental ΔG^0_{bind} values (in kcal/mol) for 20 H-G systems.The reference line represents perfect agreement between computed and experimental values.

Effect of the new radii on the $SASA$ values of the H-G complexes

Despite the fact that the values of OPT_BIND5D radii differ significantly from the common radii sets (such as Bondi), the effect of this difference on the molecular shape is relatively small. An analysis reveals that changing the radii from mbondi to OPT_BIND5D results in only about a 4% variation in the $SASA$ values of the H-G complexes.

Table 8: **Free energy components, OPT_BIND5D, 0.154 M salt:** Free energy contributions to ΔG^0_{bind} (in kcal/mol) calculated using OPT_BIND5D radii for 20 H-G systems with 0.154 M monovalent salt concentration. Further decomposition of the gas phase energies into separate terms $\Delta\Delta E_{LJ}$ and $\Delta\Delta E_{el}$ is provided in Table 16.

	Results						
H-G system	$\Delta\Delta E_{LJ} + \Delta\Delta E_{el}$	$\Delta\Delta G_{pol}$	$\Delta\Delta G_{np}$	$\Delta G_{\text{effective}}$	$-T\Delta S_{conf}$	ΔG^0_{bind}	Exp.ΔG^0_{bind}
OA-6(G4)	181.36±1.41	-197.19±1.66	-1.76±0.02	-17.59±0.29	11.33±0.20	-6.26±0.35	-9.37±0.01
OA-4(G6)	191.17±1.49	-204.56±1.70	-1.60±0.02	-14.98±0.24	9.61±0.18	-5.37±0.30	-5.34±0.01
OA-4	193.89±1.53	-209.16±1.76	-1.54±0.02	-16.81±0.25	8.32±0.18	-8.49±0.30	-6.72±0.01
α-CD-8-p	-46.57±0.73	27.75±0.53	-2.00±0.02	-20.82±0.25	13.74±0.26	-7.08±0.36	-4.62±0.02
β-CD-5-p	-19.40±0.14	8.07±0.08	-1.96±0.01	-13.28±0.10	9.85±0.25	-3.43±0.27	-4.56±0.01
OA-8(G5)	-225.24±2.26	212.50±1.99	-1.84±0.02	-14.58±0.29	9.58±0.21	-5.00±0.36	-3.72±0.01
α-CD-5-p	-13.85±0.21	5.56±0.10	-1.38±0.02	-9.67±0.15	8.04±0.24	-1.63±0.30	-2.51±0.06
β-CD-9-p	-40.81±0.59	29.33±0.45	-1.85±0.02	-13.33±0.21	11.03±0.25	-2.30±0.33	-1.70±0.05
β-CD-6-p	-29.00±0.75	23.11±0.60	-1.18±0.02	-7.07±0.19	7.16±0.26	0.09±0.42	-1.27±0.32
OA-3(G1)	193.52±1.46	-207.04±1.65	-1.58±0.02	-15.10±0.23	11.23±0.23	-3.87±0.33	-5.40±0.003
OA-5(G2)	190.68±1.37	-205.61±1.76	-1.68±0.02	-16.62±0.26	9.70±0.20	-6.92±0.33	-4.73±0.01
OA-7(G3)	-217.71±2.13	205.35±1.88	-1.79±0.02	-14.15±0.28	9.35±0.20	-4.80±0.34	-4.49±0.01
OA-1	198.77±1.48	-209.37±1.64	-1.52±0.02	-12.12±0.19	9.21±0.20	-2.91±0.28	-3.72±0.03
OA-2	198.89±1.17	-216.01±1.36	-1.73±0.02	-18.85±0.21	10.73±0.20	-8.12±0.29	-5.85±0.06
OA-3	187.77±1.30	-205.00±1.53	-1.77±0.02	-19.01±0.26	10.16±0.18	-8.85±0.32	-6.27±0.01
α-CD-6′-p	-34.76±0.56	26.98±0.47	-1.30±0.01	-9.08±0.18	8.51±0.28	-0.57±0.34	-1.51±0.04
α-CD-1-p	-5.41±0.41	5.27±0.37	-0.28±0.01	-0.42±0.06	1.59±0.70	1.17±0.72	-1.58±0.02
α-CD-7-p	-55.31±0.58	34.96±0.40	-1.84±0.01	-22.20±0.23	14.19±0.24	-8.01±0.33	-3.38±0.01
β-CD-8-p	-38.23±0.52	28.94±0.42	-1.61±0.02	-10.89±0.17	9.42±0.24	-1.47±0.32	-1.64±0.02
β-CD-3-p	-15.56±0.09	7.94±0.07	-1.71±0.01	-9.33±0.07	9.08±0.26	-0.25±0.27	-3.05±0.01

Table 9: **Free energy components, OPT_BIND5D, no salt:** Free energy contributions to ΔG^0_{bind} (in kcal/mol) calculated using OPT_BIND5D radii for 20 H-G systems with 0.0 M salt concentration. The decomposition of the gas-phase energies into the separate terms $\Delta\Delta E_{LJ}$ and $\Delta\Delta E_{el}$ is the same as shown in Table 16 , because these terms are not affected by variations in the salt concentration.

	Results						
H-G system	$\Delta\Delta E_{LJ} + \Delta\Delta E_{el}$	$\Delta\Delta G_{pol}$	$\Delta\Delta G_{np}$	$\Delta G_{\text{effective}}$	$-T\Delta S_{conf}$	ΔG^0_{bind}	Exp.G^0_{bind}
OA-6(G4)	181.36±1.41	-195.22±1.65	-1.76±0.02	-15.23 ±0.27	11.09±0.20	-4.14±0.34	-9.37±0.01
OA-4(G6)	191.17±1.46	-202.55±1.65	-1.60±0.02	-12.98±0.23	9.61±0.19	-3.37±0.30	-5.34±0.005
OA-4	193.89±1.53	-207.14±1.74	-1.54±0.02	-14.79±0.23	8.32±0.30	-6.47±0.37	-6.72±0.01
α-CD-8-p	-46.57±0.73	27.75±0.53	-2.00±0.02	-21.20±0.26	13.88±0.26	-7.32±0.37	-4.62±0.02
β-CD-5-p	-19.40±0.14	8.07±0.08	-1.96±0.01	-13.26±0.10	10.05±0.25	-3.21±0.27	-4.56±0.01
OA-8(G5)	-225.24±2.26	210.54±1.98	-1.84±0.03	-16.44±0.31	9.60±0.21	-6.84±0.37	-3.72±0.01
α-CD-5-p	-13.85±0.21	5.56±0.10	-1.38±0.02	-9.68±0.15	8.04±0.26	-1.64±0.30	-2.51±0.06
β-CD-9-p	-40.81±0.56	29.32±0.41	-1.85±0.01	-13.34±0.21	11.03±0.25	-2.31±0.33	-1.70±0.05
β-CD-6-p	-29.00±0.75	23.09±0.541	-1.18±0.02	-7.09±0.21	7.16±0.34	0.07±0.42	-1.27±0.32
OA-3(G1)	193.52±1.46	-205.00±1.63	-1.58±0.02	-13.03±0.22	10.61±0.23	-2.42±0.32	-5.40±0.003
OA-5(G2)	190.68±1.53	-203.62±1.75	-1.68±0.02	-14.45±0.25	9.26±0.20	-5.19±0.32	-4.73±0.01
OA-7(G3)	-217.71±2.13	203.42±1.87	-1.79±0.03	-16.41±0.30	10.00±0.20	-6.41±0.36	-4.49±0.01
OA-1	198.77±1.48	-207.32±1.63	-1.52±0.02	-10.01±0.17	9.14±0.20	-0.87±0.26	-3.72±0.03
OA-2	198.89±1.17	-213.95±1.35	-1.73±0.02	-16.85±0.20	8.81±0.20	-6.17±0.28	-5.85±0.06
OA-3	193.52±1.46	-205.00±1.64	-1.58±0.02	-16.90±0.25	10.29±0.18	-6.61±0.31	-6.27±0.01
α-CD-6′-p	-34.76±0.59	26.96±0.47	-1.30±0.01	-9.10±0.18	8.51±0.28	-0.59±0.34	-1.51±0.04
α-CD-1-p	-5.41±0.41	5.26±0.37	-0.28±0.01	-0.43±0.06	1.59±0.70	1.16±0.72	-1.58±0.02
α-CD-7-p	-55.31±0.58	34.95±0.40	-1.84±0.01	-22.28±0.23	14.36±0.24	-7.92±0.35	-3.38±0.01
β-CD-8-p	-38.23±0.56	28.92±0.45	-1.61±0.02	-10.91±0.17	9.42±0.26	-1.49±0.32	-1.64±0.02
β-CD-3-p	-15.56±0.12	7.93±0.07	-1.71±0.01	-9.33±0.09	9.08±0.26	-0.25±0.28	-3.05±0.33

Table 10: **Free energy components, mbondi, 0.154 M salt:** Free energy contributions to ΔG^0_{bind} (in kcal/mol) calculated using `mbondi` radii for 20 H-G systems with 0.154 M (NaCl) salt concentration.

GBNSR6 (`mbondi`)	Results						
H-G system	$\Delta\Delta E_{LJ} + \Delta\Delta E_{el}$	$\Delta\Delta G_{pol}$	$\Delta\Delta G_{np}$	$\Delta G_{\text{effective}}$	$-T\Delta S_{conf}$	ΔG^0_{bind}	Exp.ΔG^0_{bind}
OA-6(G4)	181.36±1.41	-197.40±1.66	-1.80±0.02	-17.85±0.28	11.33±0.20	-6.52±0.34	-9.37±0.01
OA-4(G6)	191.17±1.49	-201.69±1.65	-1.56±0.02	-12.08±0.20	9.61±0.19	-2.47±0.28	-5.34±0.01
OA-4	193.89±1.53	-206.22±1.72	-1.49±0.02	-13.82±0.21	8.32±0.30	-5.50±0.36	-6.72±0.01
α-CD-8-p	-46.57±0.73	27.71±0.54	-1.95±0.02	-20.81±0.24	13.74±0.26	-7.07±0.35	-4.62±0.02
β-CD-5-p	-19.40±0.14	7.61±0.08	-1.84±0.01	-13.63±0.10	9.85±0.25	-3.78±0.27	-4.56±0.01
OA-8(G5)	-225.24±2.26	213.54±2.01	-1.76±0.02	-13.46±0.27	9.58±0.21	-3.88±0.34	-3.72±0.01
α-CD-5-p	-13.85±0.20	5.75±0.11	-1.27±0.02	-9.38±0.15	8.04±0.26	-1.34±0.30	-2.51±0.06
β-CD-9-p	-40.81±0.58	30.31±0.48	-1.72±0.02	-12.22±0.21	11.03±0.25	-1.19±0.33	-1.70±0.05
β-CD-6-p	-29.00±0.75	22.76±0.58	-1.11±0.02	-7.35±0.22	7.16±0.34	-0.19±0.43	-1.27±0.32
OA-3(G1)	193.52±1.46	-204.44±1.61	-1.53±0.02	-12.46±0.19	11.23±0.23	-1.23±0.30	-5.40±0.003
OA-5(G2)	190.68±1.53	-199.29±1.68	-1.49±0.02	-10.11±0.17	9.70±0.20	-0.41±0.26	-4.73±0.01
OA-7(G3)	-217.71±2.13	205.21±1.88	-1.71±0.02	-14.21±0.28	9.35±0.20	-4.86±0.34	-4.49±0.01
OA-1	198.77±1.48	-207.58±1.62	-1.46±0.02	-10.27±0.16	9.21±0.20	-1.06±0.26	-3.72±0.03
OA-2	198.89±1.17	-213.34±1.33	-1.67±0.02	-16.12±0.18	10.73±0.20	-5.39±0.27	-5.85±0.06
OA-3	187.77±1.30	-202.64±1.50	-1.72±0.02	-16.59±0.23	10.16±0.18	-6.43±0.29	-6.27±0.01
α-CD-6′-p	-34.76±0.59	27.13±0.45	-1.26±0.01	-8.89±0.20	8.51±0.28	-0.38±0.36	-1.51±0.04
α-CD-1-p	-5.41±0.41	4.74±0.33	-0.25±0.01	-0.92±0.09	1.59±0.70	0.67±0.74	-1.58±0.02
α-CD-7-p	-55.31±0.58	35-12±0.40	-1.83±0.01	-22.02±0.21	14.19±0.24	-7.83±0.34	-3.38±0.01
β-CD-8-p	-38.23±0.56	30.76±0.44	-1.49±0.02	-8.95±0.17	9.42±0.26	0.47±0.31	-1.64±0.02
β-CD-3-p	-15.56±0.12	8.24±0.07	-1.57±0.01	-8.89±0.06	9.08±0.26	0.19±0.27	-3.05±0.01

Table 11: **Free energy components, mbondi, no salt:** Free energy contributions to ΔG^0_{bind} (in kcal/mol) calculated using `mbondi` radii for 20 H-G systems with 0.0 M salt concentration.

GBNSR6 (`mbondi`)	Results						
H-G system	$\Delta\Delta E_{LJ} + \Delta\Delta E_{el}$	$\Delta\Delta G_{pol}$	$\Delta\Delta G_{np}$	$\Delta G_{\text{effective}}$	$-T\Delta S_{conf}$	ΔG^0_{bind}	Exp.ΔG^0_{bind}
OA-6(G4)	181.36±1.41	-195.44±1.65	-1.80±0.02	-15.89±0.27	11.09±0.20	-4.80±0.34	-9.37±0.01
OA-4(G6)	191.17±1.46	-199.69±1.61	-1.56±0.02	-10.08±0.19	9.61±0.19	-0.47±0.27	-5.34±0.01
OA-4	193.89±1.53	-204.20±1.70	-1.49±0.02	-11.80±0.19	8.32±0.30	-3.48±0.34	-6.72±0.01
α-CD-8-p	-46.57±0.73	27.71±0.54	-1.95±0.02	-20.81±0.24	13.88±0.26	-6.93±0.35	-4.62±0.02
β-CD-5-p	-19.40±0.14	7.61±0.08	-1.84±0.010	-13.63±0.10	10.05±0.25	-3.58±0.27	-4.56±0.01
OA-8(G5)	-225.24±2.26	211.58±1.10	-1.76±0.03	-15.41±0.29	9.60±0.21	-5.81±0.36	-3.72±0.01
α-CD-5-p	-13.85±0.21	5.75±0.11	-1.27±0.02	-9.38±0.14	8.10±0.24	-1.34±0.26	-2.51±0.30
β-CD-9-p	-40.81±0.56	30.29±0.43	-1.72±0.01	-12.24±0.21	11.03±0.25	-1.21±0.33	-1.70±0.05
β-CD-6-p	-29.00±0.75	22.74±0.58	-1.11±0.02	-7.37±0.22	7.16±0.34	-0.21±0.43	-1.27±0.32
OA-3(G1)	193.52±1.46	-202.41±1.60	-1.53±0.02	-10.43±0.18	10.61±0.23	0.18±0.29	-5.40±0.003
OA-5(G2)	190.68±0.16	-197.30±1.66	-1.49±0.02	-8.12±0.16	9.26±0.20	1.14±0.26	-4.73±0.01
OA-7(G3)	-217.71±2.13	203.28±1.87	-1.71±0.03	-16.14±0.30	10.00±0.20	-6.14±0.36	-4.49±0.01
OA-1	198.77±1.48	-205.53±1.60	-1.46±0.02	-8.22±0.15	9.14±0.20	0.92±0.25	-3.72±0.03
OA-2	198.89±1.17	-211.25±1.32	-1.67±0.02	-14.03±0.17	8.81±0.20	-5.22±0.26	-5.85±0.06
OA-3	187.77±1.27	-200.64±1.49	-1.72±0.02	-14.26±0.22	10.29±0.18	-3.97±0.28	-6.27±0.01
α-CD-6′-p	-34.76±0.59	27.11±0.38	-1.26±0.01	-8.90±0.20	8.51±0.28	-0.39±0.36	-1.51±0.04
α-CD-1-p	-5.41±0.41	4.73±0.33	-0.25±0.01	-0.93±0.09	1.59±0.70	0.66±0.74	-1.58±0.02
α-CD-7-p	-55.31±0.58	35.12±0.40	-1.83±0.01	-22.02±0.21	14.36±0.24	-7.66±0.32	-3.38±0.01
β-CD-8-p	-38.23±0.57	30.74±0.46	-1.49±0.01	-8.98±0.16	9.42±0.26	0.44±0.31	-1.64±0.02
β-CD-3-p	-15.56±0.12	8.24±0.07	-1.57±0.01	-8.89±0.08	9.08±0.26	0.19±0.27	-3.05±0.01

Table 12: **Free energy components, PARSE, 0.154 M salt:** Free energy contributions to ΔG^0_{bind} (in kcal/mol) calculated using PARSE radii for 20 H-G systems with 0.154 M salt concentration.

GBNSR6 (PARSE)	Results						
H-G system	$\Delta\Delta E_{LJ} + \Delta\Delta E_{el}$	$\Delta\Delta G_{pol}$	$\Delta\Delta G_{np}$	$\Delta G_{\text{effective}}$	$-T\Delta S_{conf}$	ΔG^0_{bind}	Exp.ΔG^0_{bind}
OA-6(G4)	181.36±1.41	-195.92±1.64	-1.66±0.02	-16.22±0.26	11.23±0.20	-4.89±0.33	-9.37±0.01
OA-4(G6)	191.17±1.49	-199.98±1.60	-1.50±0.02	-10.31±0.18	9.61±0.19	-0.70±0.27	-5.34±0.01
OA-4	193.89±1.53	-205.54±1.71	-1.45±0.02	-13.09±0.20	8.32±0.30	-4.77±0.36	-6.72±0.01
α-CD-8-p	-46.57±0.73	32.15±0.56	-1.84±0.01	-16.26±0.21	13.74±0.26	-2.52±0.33	-4.62±0.02
β-CD-5-p	-19.40±0.14	12.25±0.10	-1.69±0.01	-8.83±0.08	9.85±0.25	1.02±0.26	-4.56±0.01
OA-8(G5)	-222.25±2.26	214.22±2.02	-1.61±0.02	-12.63±0.26	9.58±0.21	-3.05±0.33	-3.72±0.01
α-CD-5-p	-13.85±0.21	9.20±0.15	-1.19±0.02	-5.84±0.10	8.04±0.26	2.20±0.28	-2.51±0.06
β-CD-9-p	-40.81±0.57	36.31±0.49	-1.63±0.02	-6.13±0.15	11.04±0.25	4.90±0.29	-1.70±0.05
β-CD-6-p	-29.00±0.75	26.57±0.67	-1.03±0.02	-3.46±0.13	7.16±0.34	3.70±0.38	-1.27±0.32
OA-3(G1)	193.52±1.46	-203.60±1.61	-1.41±0.02	-11.49±0.18	11.23±0.23	-0.26±0.29	-5.40±0.003
OA-5(G2)	190.68±1.53	-197.64±1.66	-1.42±0.02	-8.38±0.15	9.70±0.20	1.32±0.25	-4.73±0.01
OA-7(G3)	-217.71±2.13	205.06±1.88	-1.53±0.02	-14.19±0.28	9.35±0.20	-4.84±0.34	-4.49±0.01
OA-1	198.77±1.48	-206.60±1.61	-1.37±0.02	-9.20±0.15	9.21±0.20	0.01±0.25	-3.72±0.03
OA-2	198.89±1.17	-212.46±1.32	-1.55±0.02	-15.13±0.17	10.73±0.20	-4.40±0.26	-5.85±0.06
OA-3	187.77±1.30	-201.75±1.49	-1.59±0.02	-15.57±0.22	10.16±0.18	-5.41±0.28	-6.27±0.01
α-CD-6′-p	-34.76±0.59	31.71±0.41	-1.19±0.01	-4.24±0.14	8.51±0.28	4.27±0.32	-1.51±0.04
α-CD-1-p	-5.41±0.41	5.20±0.37	-0.23±0.01	-0.44±0.06	1.59±0.70	1.15±0.72	-1.58±0.02
α-CD-7-p	-55.31±0.58	39.59±0.42	-1.72±0.01	-17.45±0.19	14.19±0.24	-3.26±0.31	-3.38±0.01
β-CD-8-p	-38.23±0.56	36.05±0.47	-1.42±0.01	-3.59±0.13	9.42±0.24	5.83±0.29	-1.64±0.02
β-CD-3-p	-15.56±0.12	11.98±0.10	-1.45±0.01	-5.04±0.07	8.71±0.26	4.04±0.26	-3.05±0.27

Table 13: **Free energy components, PARSE, no salt:** Free energy contributions to ΔG^0_{bind} (in kcal/mol) calculated using PARSE radii for 20 H-G systems with 0.0 M salt concentration

GBNSR6 (PARSE)	Results						
H-G system	$\Delta\Delta E_{LJ} + \Delta\Delta E_{el}$	$\Delta\Delta G_{pol}$	$\Delta\Delta G_{np}$	$\Delta G_{\text{effective}}$	$-T\Delta S_{conf}$	ΔG^0_{bind}	Exp.ΔG^0_{bind}
OA-6(G4)	181.36±1.41	-193.95±1.63	-1.66±0.02	-14.26±0.25	11.09±0.20	-3.17±0.32	-9.37±0.01
OA-4(G6)	191.17±1.47	-197.98±1.60	-1.50±0.02	-8.31±0.17	9.61±0.19	1.30±0.25	-5.34±0.01
OA-4	193.89±1.53	-203.52±1.70	-1.45±0.02	-11.07±0.19	8.32±0.30	2.75±0.36	-6.72±0.01
α-CD-8-p	-46.57±0.73	32.15±0.56	-1.84±0.01	-16.26±0.21	13.88±0.26	-2.38±0.33	-4.62±0.02
β-CD-5-p	-19.40±0.14	12.25±0.10	-1.69±0.01	-8.83±0.08	10.05±0.25	1.22±0.26	-4.56±0.01
OA-8(G5)	-225.24±2.29	212.27±2.03	-1.61±0.02	-14.58±0.28	9.60±0.21	-4.98±0.35	-3.72±0.01
α-CD-5-p	-13.85±0.21	9.20±0.15	-1.19±0.02	-5.84±0.10	8.04±0.26	2.20±0.28	-2.51±0.06
β-CD-9-p	-40.81±0.58	36.29±0.50	-1.63±0.02	-6.15±0.15	11.03±0.25	4.88±0.29	-1.70±0.05
β-CD-6-p	-29.00±0.75	26.55±0.67	-1.03±0.02	-3.48±0.13	7.16±0.34	3.68±0.38	-1.27±0.32
OA-3(G1)	187.77±1.30	-199.75±1.48	-1.59±0.02	-13.57±0.21	10.61±0.23	-2.96±0.31	-5.40±0.003
OA-5(G2)	190.68±1.53	-195.65±1.64	-1.42±0.02	-6.39±0.14	9.26±0.20	2.87±0.24	-4.73±0.01
OA-7(G3)	-217.71±2.13	203.13±1.87	-1.53±0.02	-16.11±0.29	10.00±0.20	-6.11±0.35	-4.49±0.01
OA-1	198.77±1.48	-204.55±1.59	-1.37±0.02	-7.15±0.14	9.14±0.20	1.99±0.24	-3.72±0.03
OA-2	198.89±1.17	-210.37±1.31	-1.55±0.01	-13.04±0.16	8.81±0.20	-4.23±0.26	-5.85±0.06
OA-3	187.77±1.30	-199.75±1.48	-1.59±0.02	-13.57±0.21	10.29±0.18	-3.28±0.28	-6.27±0.01
α-CD-6′-p	-34.76±0.59	31.69±0.51	-1.19±0.01	-4.26±0.14	8.51±0.28	4.25±0.32	-1.51±0.04
α-CD-1-p	-5.41±0.41	5.20±0.36	-0.23±0.02	-0.44±0.06	1.59±0.70	1.15±0.72	-1.58±0.02
α-CD-7-p	-55.31±0.58	39.58±0.42	-1.72±0.01	-17.45±0.19	14.36±0.24	-3.09±0.31	-3.38±0.01
β-CD-8-p	36.03±0.56	36.03±0.47	-1.42±0.01	-3.62±0.12	9.42±0.26	5.80±0.29	-1.64±0.02
β-CD-3-p	-15.56±0.12	11.97±0.08	-1.45±0.01	-5.04±0.07	9.08±0.26	4.04±0.27	-3.05±0.01

Table 14: **Free energy components, OPT_BIND (0:1), 0.154 M salt :** Free energy contributions to ΔG^0_{bind} (in kcal/mol) calculated using OPT_BIND (0:1) radii for 20 H-G systems with 0.154 M salt concentration.

GBNSR6 (OPT_BIND (0:1))	Results						
H-G system	$\Delta\Delta E_{LJ} + \Delta\Delta E_{el}$	$\Delta\Delta G_{pol}$	$\Delta\Delta G_{np}$	$\Delta G_{\text{effective}}$	$-T\Delta S_{conf}$	ΔG^0_{bind}	Exp.ΔG^0_{bind}
OA-6(G4)	181.36±1.41	-196.42±1.65	-1.90±0.02	-16.97±0.27	11.33±0.22	-5.64±0.35	-9.37±0.01
OA-4(G6)	191.17±1.49	-202.02±1.63	-1.62±0.02	-12.47±0.21	9.61±0.19	-2.86±0.29	-5.34±0.01
OA-4	193.89±1.53	-205.72±1.71	-1.54±0.02	-13.37±0.20	8.32±0.30	-5.05±0.36	-6.72±0.01
α-CD-8-p	-46.54±0.73	26.17±0.49	-2.01±0.50	-22.41±0.28	13.74±0.26	-8.67±0.38	-4.62±0.02
β-CD-5-p	-19.40±0.14	7.67±0.07	-2.00±0.01	-13.73±0.11	9.85±0.25	-3.88±0.27	-4.56±0.01
OA-8(G5)	-225.24±2.25	216.10±2.04	-1.92±0.03	-11.06±0.25	9.58±0.21	-1.48±0.33	-3.72±0.01
α-CD-5-p	-13.85±0.21	5.33±0.09	-1.42±0.02	-9.95±0.15	8.04±0.24	-1.91±0.31	-2.51±0.06
β-CD-9-p	-40.81±0.58	30.32±0.45	-1.80±0.02	-12.29±0.21	11.03±0.25	-1.26±0.33	-1.70±0.05
β-CD-6-p	-29.00±0.75	22.46±0.58	-1.18±0.02	-7.72±0.21	7.16±0.34	-0.56±0.43	-1.27±0.32
OA-3(G1)	193.52±1.46	202.24±1.59	-1.62±0.02	-10.35±0.17	11.23±0.23	0.88±0.29	-5.40±0.003
OA-5(G2)	190.68±1.53	-202.72±1.73	-1.68±0.02	-13.72±0.22	9.70±0.20	-4.02±0.30	-4.73±0.01
OA-7(G3)	-217.71±2.13	207.41±1.91	-1.88±0.02	-12.17±0.26	9.35±0.20	-2.82±0.33	-4.49±0.01
OA-1	198.77±1.48	-205.65±1.60	-1.51±0.02	-8.39±0.14	9.21±0.20	0.82±0.24	-3.72±0.03
OA-2	198.89±1.17	-211.90±1.32	-1.74±0.02	-14.76±0.17	10.73±0.20	-4.03±0.26	-5.85±0.06
OA-3	187.77±1.30	-200.94±1.49	-1.83±0.02	-14.99±0.21	10.16±0.18	-4.83±0.28	-6.27±0.01
α-CD-6′-p	-34.76±0.59	26.76±0.45	-1.30±0.01	-9.29±0.19	8.51±0.28	-0.78±0.35	-1.51±0.04
α-CD-1-p	-5.41±0.41	5.14±0.36	-0.29±0.02	-0.55±0.07	1.59±0.70	1.04±0.72	-1.58±0.02
α-CD-7-p	-55.31±0.58	32.85±0.36	-1.84±0.01	-24.30±0.25	14.19±0.24	-10.11±0.35	-3.38±0.01
β-CD-8-p	-38.23±0.56	30.34±0.45	-1.54±0.02	-9.42±0.17	9.41±0.26	0.01±0.31	-1.64±0.02
β-CD-3-p	-15.56±0.09	7.62±0.06	-1.75±0.01	-9.69±0.09	9.08±0.26	-0.61±0.28	-3.05±0.01

Table 15: **Free energy components, OPT_BIND (0:1), no salt :** Free energy contributions to ΔG^0_{bind} (in kcal/mol) calculated using OPT_BIND (0:1) radii for 20 H-G systems with 0.0 M salt concentration.

GBNSR6 (OPT_BIND (0:1))	Results						
H-G system	$\Delta\Delta E_{LJ} + \Delta\Delta E_{el}$	$\Delta\Delta G_{pol}$	$\Delta\Delta G_{np}$	$\Delta G_{\text{effective}}$	$-T\Delta S_{conf}$	ΔG^0_{bind}	Exp.ΔG^0_{bind}
OA-6(G4)	181.36±1.41	-194.46±1.64	-1.90±0.02	-15.01±0.26	11.09±0.20	-3.92±0.33	-9.37±0.01
OA-4(G6)	191.17±1.46	-200.02±1.62	-1.62±0.02	-10.47±0.20	9.61±0.19	-0.86±0.28	-5.34±0.005
OA-4	193.89±1.53	-203.70±1.70	-1.54±0.02	-11.35±0.29	8.32±0.30	-3.03±0.36	-6.72±0.01
α-CD-8-p	-46.57±0.73	26.17±0.49	-2.01±0.02	-22.41±0.28	13.88±0.26	-8.53±0.38	-4.62±0.02
β-CD-5-p	-19.40±0.14	7.67±0.07	-2.00±0.01	-13.73±0.11	10.05±0.25	-3.68±0.27	-4.56±0.01
OA-8(G5)	-225.24±2.26	214.14±2.03	-1.92±0.03	-13.02±0.26	9.60±0.21	-3.42±0.33	-3.72±0.01
α-CD-5-p	-13.85±0.21	5.32±0.09	-1.42±0.02	-9.95±0.15	8.04±0.24	-1.91±0.31	-2.51±0.06
β-CD-9-p	-40.81±0.57	30.31±0.43	-1.80±0.01	-12.31±0.20	11.03±0.26	-1.28±0.33	-1.70±0.05
β-CD-6-p	-29.00±0.75	22.44±0.58	-1.18±0.02	-7.74±0.22	7.16±0.34	-0.58±0.43	-1.27±0.32
OA-3(G1)	193.52±1.46	-200.21±1.58	-1.62±0.019	-8.32±0.16	10.61±0.23	2.29±0.28	-5.40±0.003
OA-5(G2)	190.68±1.53	-200.73±1.71	-1.68±0.02	-11.73±0.22	9.26±0.20	-2.47±0.30	-4.73±0.01
OA-7(G3)	-217.71±2.13	205.48±1.90	-1.88±0.03	-14.11±0.27	10.00±0.2	-4.11±0.34	-4.49±0.01
OA-1	198.77±1.48	-203.61±1.58	-1.51±0.02	-6.35±0.13	9.14±0.20	2.79±0.24	-3.72±0.03
OA-2	198.89±1.17	-209.81±1.31	-1.74±0.02	-12.67±0.16	8.81±0.20	-3.86±0.26	-5.85±0.06
OA-3	187.77±1.30	-198.94±1.48	-1.83±0.02	-13.00±0.20	10.29±0.18	-2.71±0.27	-6.27±0.01
α-CD-6′-p	-34.76±0.59	26.74±0.45	-1.30±0.02	-9.31±0.19	8.51±0.28	-0.80±0.35	-1.51±0.04
α-CD-1-p	-5.41±0.41	5.14±0.36	-0.29±0.02	-0.56±0.07	1.59±0.70	1.03±0.72	-1.58±0.02
α-CD-7-p	-55.31±0.58	32.84±0.36	-1.84±0.01	-24.30±0.25	14.36±0.24	-9.94±0.35	-3.38±0.01
β-CD-8-p	-38.23±0.56	30.32±0.45	-1.54±0.01	-9.44±0.17	9.42±0.26	-0.02±0.31	-1.64±0.02
β-CD-3-p	-15.56±0.12	7.62±0.06	-1.75±0.01	-9.69±0.09	9.08±0.26	-0.61±0.28	-3.05±0.01

Table 16: **Free energy components, OPT_BIND5D, 0.154 M salt (with decomposition of the gas phase energies):** Free energy contributions to ΔG^0_{bind} (in kcal/mol) calculated using OPT_BIND5D radii for 20 H-G systems with 0.154 M monovalent salt concentration.

	Results							
H-G system	$\Delta\Delta E_{LJ}$	$\Delta\Delta E_{el}$	$\Delta\Delta G_{pol}$	$\Delta\Delta G_{np}$	$\Delta G_{\text{effective}}$	$-T\Delta S_{conf}$	ΔG^0_{bind}	Exp.ΔG^0_{bind}
OA-6(G4)	-22.01±0.29	203.36±1.70	-197.19±1.66	-1.76±0.02	-17.59±0.29	11.33±0.20	-6.26±0.35	-9.37±0.01
OA-4(G6)	-18.65±0.25	209.82±1.73	-204.56±1.70	-1.60±0.02	-14.98±0.24	9.61±0.18	-5.37±0.30	-5.34±0.01
OA-4	-17.45±0.22	211.34±1.75	-209.16±1.76	-1.54±0.02	-16.81±0.25	8.32±0.18	-8.49±0.30	-6.72±0.01
α-CD-8-p	-16.68±0.16	-29.89±0.72	27.75±0.53	-2.00±0.02	-20.82±0.25	13.74±0.26	-7.08±0.36	-4.62±0.02
β-CD-5-p	-17.68±0.10	-1.72±0.08	8.07±0.08	-1.96±0.01	-13.28±0.10	9.85±0.25	-3.43±0.27	-4.56±0.01
OA-8(G5)	-18.10±0.27	-207.14±1.98	212.50±1.99	-1.84±0.02	-14.58±0.29	9.58±0.21	-5.00±0.36	-3.72±0.01
α-CD-5-p	-12.80±0.18	-1.06±0.07	5.56±0.10	-1.38±0.02	-9.67±0.15	8.04±0.24	-1.63±0.30	-2.51±0.06
β-CD-9-p	-13.53±0.15	-27.29±0.51	29.33±0.45	-1.85±0.02	-13.33±0.21	11.03±0.25	-2.30±0.33	-1.70±0.05
β-CD-6-p	-7.89±0.20	-21.11±0.61	23.11±0.60	-1.18±0.02	-7.07±0.19	7.16±0.26	0.09±0.42	-1.27±0.32
OA-3(G1)	-17.72±0.22	211.24±1.67	-207.04±1.65	-1.58±0.02	-15.10±0.23	11.23±0.23	-3.87±0.33	-5.40±0.003
OA-5(G2)	-17.74±0.25	208.42±1.76	-205.61±1.76	-1.68±0.02	-16.62±0.26	9.70±0.20	-6.92±0.33	-4.73±0.01
OA-7(G3)	-17.29±0.26	-200.42±1.87	205.35±1.88	-1.79±0.02	-14.15±0.28	9.35±0.20	-4.80±0.34	-4.49±0.01
OA-1	-14.37±0.18	213.14±1.65	-209.37±1.64	-1.52±0.02	-12.12±0.19	9.21±0.20	-2.91±0.28	-3.72±0.03
OA-2	-18.88±0.18	217.77±1.34	-216.01±1.36	-1.73±0.02	-18.85±0.21	10.73±0.20	-8.12±0.29	-5.85±0.06
OA-3	-18.99±0.22	206.76±1.51	-205.00±1.53	-1.77±0.02	-19.01±0.26	10.16±0.18	-8.85±0.32	-6.27±0.01
α-CD-6′-p	-10.74±0.16	-24.02±0.52	26.98±0.47	-1.30±0.01	-9.08±0.18	8.51±0.28	-0.57±0.34	-1.51±0.04
α-CD-1-p	-1.81±0.14	-3.60±0.29	5.27±0.37	-0.28±0.01	-0.42±0.06	1.59±0.70	1.17±0.72	-1.58±0.02
α-CD-7-p	-14.07±0.11	-41.25±0.59	34.96±0.40	-1.84±0.01	-22.20±0.23	14.19±0.24	-8.01±0.33	-3.38±0.01
β-CD-8-p	-12.55±0.16	-25.68±0.47	28.94±0.42	-1.61±0.02	-10.89±0.17	9.42±0.24	-1.47±0.32	-1.64±0.02
β-CD-3-p	-13.81±0.08	-1.75±0.08	7.94±0.07	-1.71±0.01	-9.33±0.07	9.08±0.26	-0.25±0.27	-3.05±0.01

References

(1) Forouzesh, N.; Onufriev, A. V. MMGB/SA Consensus Estimate of the Binding Free Energy Between the Novel Coronavirus Spike Protein to the Human ACE2 Receptor. *bioRxiv* **2020**,

(2) Mukhopadhyay, A.; Fenley, A.; Tolokh, I.; Onufriev, A. Charge hydration asymmetry: the basic principle and how to use it to test and improve water models. *The Journal of Physical Chemistry B* **2012**, *116*, 9776–9783.

(3) Mukhopadhyay, A.; Tolokh, I.; Onufriev, A. Accurate Evaluation of Charge Asymmetry in Aqueous Solvation. *J. Phys. Chem. B* **2015**, *119*, 6092–6100.

(4) Muddana, H.; Sapra, N.; Fenley, A.; Gilson, M. The electrostatic response of water to neutral polar solutes: Implications for continuum solvent modeling. *The Journal of Chemical Physics* **2013**, *138*, 224504.
